# Alteration of oxidative-stress and related marker levels in mouse colonic tissues and fecal microbiota structures with chronic ethanol administration: Implications for the pathogenesis of ethanol-related colorectal cancer

**DOI:** 10.1371/journal.pone.0246580

**Published:** 2021-02-12

**Authors:** Hideo Ohira, Atsuki Tsuruya, Daiki Oikawa, Wao Nakagawa, Rie Mamoto, Masahira Hattori, Toshiyuki Waki, Seiji Takahashi, Yoshio Fujioka, Toru Nakayama

**Affiliations:** 1 Division of Clinical Nutrition, Faculty of Nutrition, Kobe Gakuin University, Kobe, Japan; 2 Department of Biomolecular Engineering, Graduate School of Engineering, Tohoku University, Sendai, Japan; 3 Graduate School of Advanced Science and Engineering, Waseda University, Tokyo, Japan; Toho University Graduate School of Medicine, JAPAN

## Abstract

Chronic ethanol consumption is a risk factor for colorectal cancer, and ethanol-induced reactive oxygen species have been suggested to play important roles in the pathogenesis of ethanol-related colorectal cancer (ER-CRC). In this study, the effects of 10-week chronic administration of ethanol on the colonic levels of oxidative stress and advance glycation end product (AGE) levels, as well as fecal microbiota structures, were examined in a mouse model. Chronic oral administration of ethanol in mice (1.0 mL of 1.5% or 5.0% ethanol (v/v) per day per mouse, up to 10 weeks) resulted in the elevation of colonic levels of oxidative stress markers (such as 8-hydroxy-2’-deoxyguanosine and 4-hydroxynonenal) compared to control mice, and this was consistently accompanied by elevated levels of inflammation-associated cytokines and immune cells (Th17 and macrophages) and a decreased level of regulatory T (Treg) cells to produce colonic lesions. It also resulted in an alteration of mouse fecal microbiota structures, reminiscent of the alterations observed in human inflammatory bowel disease, and this appeared to be consistent with the proposed sustained generation of oxidative stress in the colonic environment during chronic ethanol consumption. Moreover, the first experimental evidence that chronic ethanol administration results in elevated levels of advanced glycation end products (AGEs) and their receptors (RAGE) in the colonic tissues in mice is also shown, implying enhanced RAGE-mediated signaling with chronic ethanol administration. The RAGE-mediated signaling pathway has thus far been implicated as a link between the accumulation of AGEs and the development of many types of chronic colitis and cancers. Thus, enhancement of this pathway likely exacerbates the ethanol-induced inflammatory states of colonic tissues and might at least partly contribute to the pathogenesis of ER-CRC.

## Introduction

Chronic ethanol consumption has been shown to be one of the major risk factors for colorectal cancer (CRC) [1, 2]. Previous colonoscopic screening results of Japanese alcoholic men, who are chronic ethanol consumers, showed colorectal adenoma in 54.5% of these subjects and intramucosal and invasive CRCs in 5.9% [3]. The mechanism by which chronic ethanol consumption increases CRC risk has been under examination. Chronic ethanol consumers retain ethanol in their blood for prolonged periods, which equilibrates between blood and tissues [4]. It has been proposed that ethanol and its metabolites promote inflammation and oxidative stress of the colon and rectum via multiple pathways [2, 5, 6], which leads to CRC. In the colorectal epithelial cells, ethanol oxidation, coupled with the mitochondrial electron transport system, coincidentally gives rise to the formation of reactive oxygen species (ROS) [7, 8]. Inflammation and ROS may cause DNA damage that initiates their malignant transformation [2, 5–8] and lipid peroxidation that mediates the formation of carcinogenic etheno-DNA adducts [9]. However, there are few studies using animal models that have examined the effect of long-term chronic administration of ethanol on the pathophysiological processes in the gut [10], although there are numerous animal studies about its effect on hepatic disorders (see [11] for example).

In 2016, we analyzed and compared the gut microbiota structures of alcoholic and non-alcoholic people to identify the ecophysiological consequences of alcoholism on human gut microbiota [12]. The results showed that the gut microbiotas of alcoholics were in a dysbiosis state, in which obligate anaerobes (such as *Bacteroides* and ruminococci) decreased, and facultative anaerobes (such as streptococci and bacterial species belonging to Enterobacteriaceae) were enriched. Because obligate anaerobes are generally more susceptible to ROS than facultative anaerobes, these observations were consistent with the proposed sustained formation of ROS and ROS-induced oxidative stress in the colonic environment during chronic ethanol consumption. The effects of chronic oral administration of ethanol on colonic oxidative-stress and inflammatory markers remain to be clarified in detail; thus, examination of these aspects would provide important information for insights into the pathogenesis of ethanol-related CRC (ER-CRC).

Advanced glycation end products (AGEs) are a group of heterogeneous compounds formed via non-enzymatic glycation/glycoxidation of proteins [13, 14]. Increased accumulation of AGEs is at least in part related to ROS-induced oxidative stress. AGEs bind to their specific receptors (RAGE) to mediate diverse cellular responses, which include multiple pathological effects associated with oxidative stress and inflammation via downstream signaling and transcriptional activation, inducing chronic inflammatory diseases (*e*.*g*., inflammatory bowel diseases (IBDs)) [14–16], genomic instability, and cancer initiation [17]. However, the extent that chronic consumption of ethanol contributes to the accumulation of AGEs in the colorectal tissues in humans remained to be examined, and, thus, the role of colorectal AGEs and RAGE, if any, in the pathogenesis of ethanol-related CRC (ER-CRC) has been unclear.

In this study, to obtain insights into the ROS-related pathogenesis of ER-CRC, the effects of chronic ethanol administration on the levels of colonic oxidative-stress and inflammatory markers and AGEs/RAGE, as well as fecal microbiota structures, were comprehensively investigated in mice.

## Materials and methods

### Animals

Prior to the initiation of this work, all animal-related experiments were approved by the Ethical Committee for Animal Experimentation of Kobe Gakuin University, Kobe, Japan (approval number, A17-54). As required by the Guidelines for Proper Conduct of Animal Experiments by the Science Council of Japan, methods and procedures were used to minimize pain and/or distress of all animals used in this study.

C57BL/6NCr male mice (6–7 weeks old) were obtained from SLC (Hamamatsu, Japan). Upon arrival, mice had an average weight of 21.5 ± 1.4 g at 6–7 weeks of age. All mice were housed, one mouse per cage, in an animal breeding room of SPF level, Animal Experimentation Facility, Kobe Gakuin University, under standard conditions [23–24°C, 12 h light/dark cycle (lights on from 8 a.m. to 8 p.m.)]. The Animal Experimentation Facility undertakes a twice a year health screening, covering various bacterial, viral, and parasitic organisms, and the obtained colony screened negative for all of them. Food and water were available *ad libitum*. Upon arrival and throughout the study, mice were fed a defined AIN93 (M) rodent chow (SLC) to control the amount of phytochemicals in their diet.

### Experimental design

Three groups of 8-week-old mice (8 mice/group per experiment) were randomly assigned to one of three groups, and each group was orally gavaged daily with one of the following: 1.0 mL of water (control group); 1.0 mL of 1.5% ethanol (v/v), which corresponded to an ethanol dosage of 0.5–0.6 g ethanol/kg body weight/day (1.5% ethanol group); and 1.0 mL of 5.0% ethanol (v/v), which corresponded to an ethanol dosage of 1.7–2.0 g ethanol/kg body weight/day (5.0% ethanol group). Sample sizes (n = 8) were based on a previous report of a chronic ethanol administration experiment [18]; the required sample number for each analysis was calculated, and a minimum sample size of n = 4-5/group was found to be necessary for sufficient statistical power.

To evaluate the continuous intake of a fixed amount of ethanol, gavage was performed once per day using a FG5202K (silicon 20-gauge) disposable feeding needle (AS ONE, Osaka, Japan) from the beginning at 8 weeks of age until the mice reached 10 weeks of age (2-wk subjects; 24 mice, 8 mice per group) or 18 weeks of age (10-wk subjects; 24 mice, 8 mice per group) (see S1 Fig for experimental design). Gavage was conducted in the laboratory room of SPF level in the above-mentioned facility. It should be noted that one 10-wk subject of the 5.0% EtOH group appeared to show severe clinical wasting and was removed from the study at week 4 of the experiment to minimize pain and discomfort according to the humane endpoints.

At the experimental endpoints (*i*.*e*., week 2 and week 10), the mice were anesthetized by their initial exposure to 4% isoflurane (FUJIFILM Wako Pure Chemical, Osaka, Japan) using an anesthesia vaporizer (Muromachi Kikai, Tokyo, Japan) followed by sustained exposure to 3% isoflurane. Mice were sacrificed painlessly and subjected to necropsy. Blood was collected from the abdominal aorta, and separate sections of each liver, cecum, and colon were excised, washed, and stored for histological, biochemical, enzyme-linked immunosorbent assays (ELISAs), and gene expression analyses.

### Biochemical analysis

Blood samples were treated with 10 IU/mL heparin sodium (Nacalai Tesque, Kyoto, Japan). Plasma was obtained by centrifuging the blood at 1000 x *g* for 15 min at 4°C. Activities of plasma alanine aminotransferase (ALT) and aspartate aminotransferase (AST), as well as plasma triacylglycerol (TG), were measured using kits (L-Type Wako ALT, L-Type Wako AST, and L-Type Wako TG M, respectively), each obtained from FUJIFILM Wako Pure Chemical.

### Liver tissue lipid levels

Liver homogenate was prepared at 4°C with a kit (Lipid Droplet Isolation kit; Cell Biolabs, San Diego, CA, USA) using a Polytron homogenizer (model 1300D; Central Scientific Commerce, Tokyo, Japan), in which tissues were homogenized 5 times using a loose pestle for 10 sec each time, followed by homogenization using a tight pestle for 5 times for 10 sec each time. The resulting homogenate was centrifuged at 20,000 x *g* at 4°C for 3 h. Triglycerides and proteins in the supernatant were determined using kits [L-type Wako TG M and protein assay reagents (Bio-Rad Laboratories, Hercules, CA, USA), respectively] according to the manufacturers’ guidelines.

### Histology

Tissue samples from the liver, the small intestine, and the colon were fixed overnight in 4% (v/v) neutral formaldehyde solution (Nacalai Tesque) and embedded in paraffin. Tissue slices (4 μm in thickness) were subjected to the following staining: hematoxylin/eosin (HE; for liver, small intestine, colon), Oil red O (for liver), and Toluidine Blue (TB; for small intestine and colon). These procedures were outsourced to Kyodo Byori (Kobe, Japan). Finally, these histology specimens were observed using a model VH-8000 microscope (KEYENCE, Osaka, Japan).

### Microscopic evaluation of colonic lesions

For each animal, five parts were randomly taken from colonic tissue section samples and subjected to microscopic evaluation. Semi-quantitative degrees of inflammation of the colonic lumen were graded from 0 (the absence of inflammation) to 11 (the severest inflammation) according to the sum of the scores based on the following observations: (a) loss of mucosa architecture (score, 0–3); (b) cellular infiltration (score, 0–3); (c) muscle thickening (score, 0–3); (d) crypt abscess formation (score, 0–1); and (e) goblet cell depletion (score, 0–1) [19, 20].

### Immunohistochemical and immunofluorescence staining

Slices (4 μm in thickness; see above) of colonic tissue sections were placed on clean, positively charged microscope slide glasses and dried by heating for 2 h at 60°C in a tissue-drying oven. The section slices were deparaffinized by washing in xylene for 5 min, 3 times, and dehydrated in a series of graded concentrations of ethanol. For heat-induced antigen retrieval, the slide glasses were heated at 100°C for 20 min in 10 mM sodium citrate buffer, pH 6.0, and then cooled to room temperature in the same buffer for 20 min, followed by washing with 1x Tris-buffered saline containing 0.1% Tween 20 at room temperature for 1 min.

Immunohistochemical staining showed that endogenous peroxidase activity was blocked by incubation of the slices with 3% H_2_O_2_ for 5 min. The slices were then incubated with 2% bovine serum albumin in phosphate buffered saline for 30 min, followed by incubation at 4°C overnight with one of the following primary antibodies: rabbit anti-intestinal mucin 2 (MUC2) polyclonal antibody (Abcam, Cambridge, UK), rabbit anti-8-hydroxy-2’-deoxyguanosine (8-OHdG) monoclonal antibody (Abcam), rabbit anti-AGE polyclonal antibody (Abcam), rabbit anti-RAGE polyclonal antibody (Abcam), rabbit anti-nitrotyrosine monoclonal antibody (Zymed Laboratories Inc, South San Francisco, CA, USA), and rabbit anti-4-hydroxynonenal (4-HNE) monoclonal antibody (NIKKEN SEIL, Tokyo, Japan). Subsequently, the slices were incubated with the biotinylated goat anti-rabbit IgG antibody (Abcam) for 20 min at room temperature. Immune complexes were visualized by peroxidase-catalyzed oxidation of diaminobenzidine (Dojindo Laboratories, Kumamoto, Japan), and the sections were counterstained with hematoxylin. These procedures were outsourced to Kyodo Byori. Immuno-positive cells were observed by microscopic examination in randomly selected high-power fields (400X) of these histology specimens using a model VH-8000 microscope (KEYENCE).

The deparaffinized section slices (see above) were also used for immunofluorescence detection of retinoic acid receptor-related orphan receptor-γt (RORγt), forkhead box protein P3 (Foxp3), F4/80 (F4/80 refers to a 160-kD glycoprotein expressed by murine macrophages), and inducible nitric oxide synthase (iNOS), as follows. The slices were incubated with 2% bovine serum albumin in phosphate buffered saline for 30 min, followed by incubation at 4°C overnight with one of the following primary antibodies: rat monoclonal anti-mouse RORγt antibody (Abcam), rat monoclonal anti-mouse FoxP3 antibody (eBioscience, San Diego, CA, USA), rabbit monoclonal anti-mouse CD4 antibody (Abcam), rabbit polyclonal anti-mouse iNOS antibody (GeneTex, Irvine, CA, USA), and rat monoclonal anti-mouse F4/80 antibody (BMA Biomedicals, August, Switzerland). Subsequently, the slices were incubated with goat anti-rabbit Alexa Fluor 488 (for rabbit primary antibodies) or goat anti-rat Alexa Fluor 568 (for rat primary antibodies) (both from Thermo Fisher Scientific, Waltham, MA, USA). 4′,6-Diamidino-2-phenylindole (DAPI) (Thermo Fisher Scientific) was used to stain nuclei. Isotype controls were used for all assays. These procedures were outsourced to Kyodo Byori. Immuno-positive cells were observed by microscopic examination in randomly selected high-power fields (400X) of these histology specimens using a model BZ-X700 fluorescence microscope (KEYENCE).

### Flow cytometry analysis

Mouse colonic tissues were cut into small fragments and shaken in Hank’s balanced salt solution containing 10% fetal bovine serum and 5 mM EDTA at 37°C for 25 min. These small fragments were them subjected to treatment with Tissue (Tumor) Dissociation/Single Cell isolation kit (101 Bio LLC, CA, USA) for 10 min according to the manufacturer’s guidelines. The cells were collected by centrifugation and stored in BAMBANKER (GC LYMPHOTEC, Tokyo, Japan) in liquid nitrogen.

Single cell suspensions were pre-incubated with purified rat anti-mouse CD16/CD32 (FcγRIII/FcγRII) blocking antibody (BD Biosciences, San Jose, CA, USA) for 10 min on ice, followed by incubation for 30 min at 4°C with a combination of the following fluorescence-labeled rat anti-mouse monoclonal antibodies: CD11b-FITC (Abcam) and F4/80-PE (BioLegend, San Diego, CA, USA).

CD4^+^ cells were purified from the isolated single cells by positive selection using MACS SmartStrainers (70 μm) and a MiniMACS Separator Kit (Miltenyi Biotec, Bergisch Glabach, Germany). For staining of intracellular Foxp3, cells were subjected to treatment with a FlowX FoxP3 Fixation & Permeabilization Buffer kit (R&D Systems, Minneapolis, MN, USA) according to the manufacturer’s instructions. CD4^+^ cells were incubated with blocking antibody (BD Bioscience) for 10 min on ice, followed by incubation with the following rat monoclonal antibodies: anti-mouse CD4-FITC antibody (BD Biosciences), anti-Foxp3-PE antibody (BioLegend), and ant-RORγt-PE antibody (Thermo Fisher Scientific), anti-CD45-PECy7 antibody (BioLegend). Dead cells were excluded after staining them with 7-aminoactinomycin D (7-AAD) (BioLegend).

Cells were analyzed using a BD FACSCanto flow cytometer (BD Biosciences), and the results were analyzed with BD FACSDiva software (BD Biosciences).

Cells were chilled on ice until fluorescence activated cell sorting. The following gating strategies were used to identify specific cell populations (see also S2 Fig). The CD45^+^ mature leukocyte populations were identified by CD45-side scatter gating, in which dead cells that were stained with 7-AAD were excluded. The CD45^+^ CD4^+^ T lymphocytes were gated with anti-RORγt-PE and anti-Foxp3-PE to identify Th17 cells and Treg cells. The CD45^+^ CD11b^+^ cell populations were gated with F4/80-PE to identify macrophages. Two-parameter density plots were used to monitor changes in relative abundance of CD45^+^ specific cells populations.

### ELISA

Colonic tissue samples were homogenized with M-PER Mammalian Protein Extraction Reagent (Pierce, Rockford, IL, USA) supplemented with a recommended dilution of a protease inhibitor cocktail (Nacalai Tesque) using a homogenizer (see above), followed by centrifugation at 4°C. The protein concentration of the supernatant was determined using a protein assay reagent (Bio-Rad Laboratories). DNA was isolated from the supernatant using a DNAzol Reagent (Thermo Fisher Scientific) according to the manufacturer’s guidance. The levels of 8-OHdG, 3-nitrotyrosine, 4-HNE, Foxp3, AGEs, and RAGE in the supernatant were respectively quantified using the following ELISA kits: OxiSelect oxidative DNA damage ELISA kit (Cell Biolabs), OxiSelect Nitrotyrosine ELISA kit (Cell Biolabs), OxiSelect HNE adduct competitive ELISA kit (Cell Biolabs), FoxP3 ELISA Kit (CLOUD-CLONE, Katy, TX, USA), OxiSelect AGE Competitive ELISA Kit, and Mouse RAGE Quantikine ELISA Kit (R&D Systems).

### qRT-PCR

Total RNA was extracted from colonic tissue samples using TRIzol Reagent (for RNA extraction; Invitrogen). First strand cDNA was synthesized at 37°C for 60 min from 1 μg of total RNA using a ReverTra Ace-α first strand cDNA synthesis kit (Toyobo, Osaka, Japan) using a thermal cycler (Gene Amp PCR System model 9700; Applied Biosystems, Carlsbad, CA). The mixture was then incubated at 94°C for 5 min to inactivate the enzyme, followed by incubation at 4°C. To quantify *tumor necrosis factor-α* (*TNF-α*), *interleukin-6* (*IL-6*), *interleukin 17A* (*IL-17A*), *monocyte chemotactic protein-1* (*MCP-1*), and *β-actin* mRNAs, quantitative reverse transcription-PCR (qRT-PCR) was performed using a LightCycler System (Roche Diagnostics, Mannheim, Germany) and LightCycler Fast Start DNA Master Plus SYBR Green 1 (Roche Diagnostics). Thermal cycling conditions were as follows: 45 cycles at 95°C for 10 sec, 60°C for 10 sec, and 72°C for 10–20 sec. Nucleotide sequences of qRT-PCR primers are listed in S1 Table.

### Sequencing of bacterial 16S rRNA gene amplicons, processing raw data, and data analysis

#### DNA isolation from mouse feces

Mouse feces (4 mice/group) were collected at weeks 2 and 5 by means of fecal disimpaction. The collected fecal samples were immediately mixed with an appropriate volume of RNAprotect Bacteria Reagent (Qiagen, Tokyo Japan) and stored at –80°C until analysis. Bacterial genomic DNAs were extracted as follows. After thawing, the fecal samples were subjected to centrifugation at 8000 x *g* for 10 min. The precipitate was suspended in 200 μl of 10 mM Tris-HCl/1 mM EDTA (TE) buffer, pH 7.0, containing 15 mg/mL lysozyme (Sigma-Aldrich, St. Louis, MO, USA). After incubation of the mixture at 37°C for 1 h, 200 μl of achromopeptidase (4000 units/mL, FUJIFILM Wako Pure Chemical) were added, and the mixture was further incubated at 37°C for 30 min. Subsequently, 400 μl of a mixture containing 2 mg/mL of proteinase K and 2% sodium dodecyl sulfate were added, and the resulting mixture was incubated at 55°C for 1 h. The mixture was then treated with phenol/chloroform/isoamyl alcohol (Life Technologies Japan, Tokyo, Japan), followed by treatment with RNase A (FUJIFILM Wako Pure Chemical), as described previously [12]. DNA was precipitated by adding an equal volume of a mixture of 20% polyethylene glycol 6000 and 2.5 M NaCl followed by centrifugation at 8000 x *g* at 4°C, washed with 75% ethanol, and dissolved in TE.

#### 16S rRNA sequencing, processing of raw sequence data, and data analyses

The 16S rRNA amplicon sequences were deposited in DDBJ under the accession number DRA010530. The V1-V2 region of the 16S rRNA gene was amplified using a 27 modFw and 338Rv primers (for nucleotide sequences of primers, see ref [12]). The PCR amplicons were pyrosequenced as described previously [12]. Processing of raw sequence data and data analyses [operational taxonomic Unit (OTU) analysis and UniFrac distance analysis] were completed as described previously [12]. In the OTU analysis, the 16S reads were clustered with a pair-wise identity cutoff of 96%. Sequence similarity thresholds of 70% and 97% were applied to the phylum and genus assignments, respectively.

### Statistical analysis

Data were assessed using a paired *t*-test, Welch’s *t* test for comparisons between two groups or ANOVA with Tukey-Kramer post-hoc comparisons, and were expressed as mean ± SD. Statistical analysis was performed using GraphPad Prism ver 6.0 (GraphPad Software Inc., San Diego, CA). Two-tailed values of p< 0.05 were considered significant.

## Results

### Characterization of mice with chronic oral administration of ethanol

#### Body weight and liver functions

**Fig 1:**
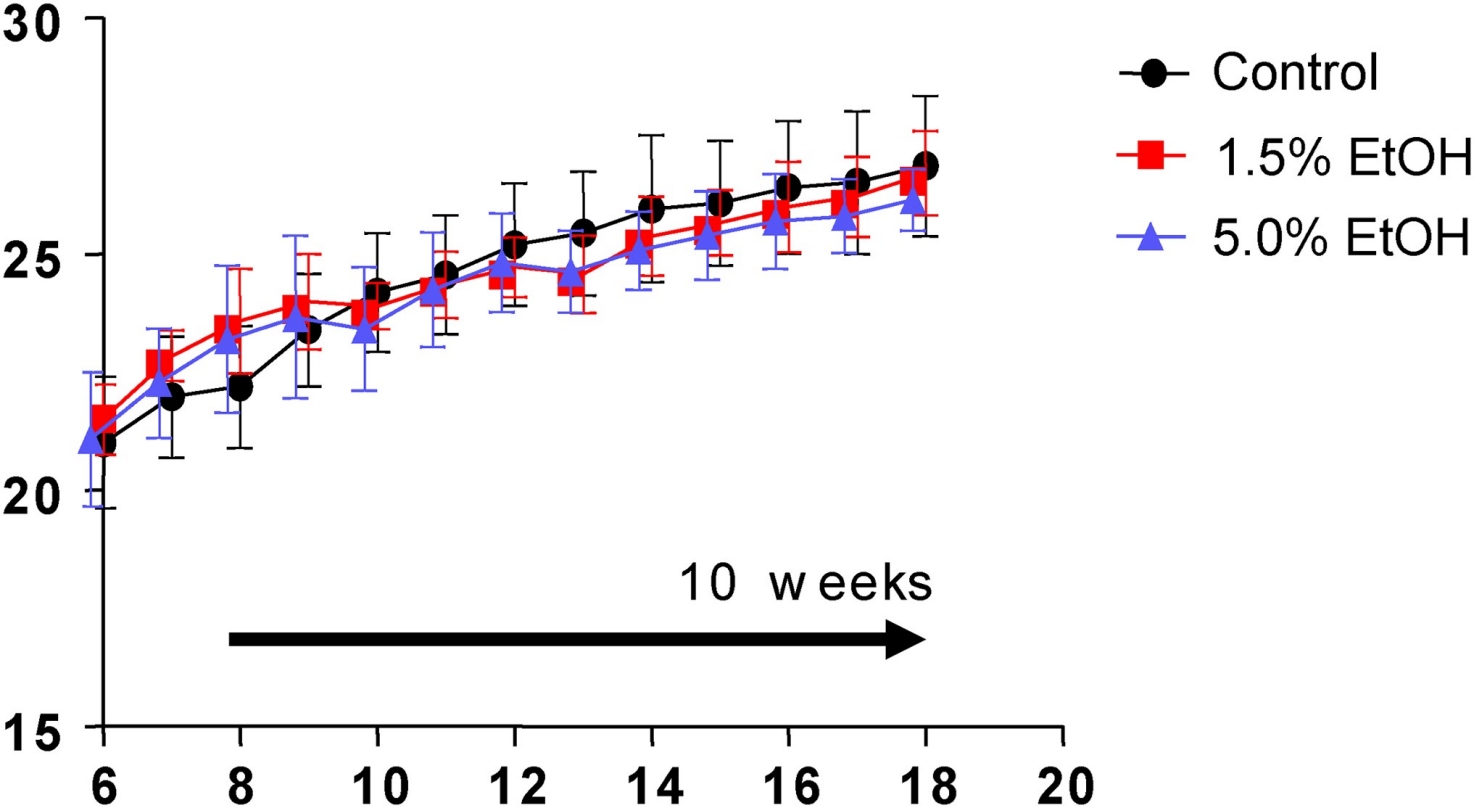
Course of body weight changes during the experiment. Eight-week-old mice were orally gavaged daily with 1.0 mL/day of water (control, black circles), 1.0 mL/day of 1.5% (v/v) ethanol (the 1.5%-ethanol group, red squares), and 1.0 mL/day of 5.0% (v/v) ethanol (the 5.0%-ethanol group, blue triangles) for 10 weeks (horizontal arrow). Body weights were measured every week. The data are expressed as means ±SD (n = 7 to 8) for each group and assessed using ANOVA with the Tukey-Kramer test.

Eight-week-old mice were orally gavaged daily for 10 weeks with 1.0 mL of water (the control group), 1.0 mL of 1.5% ethanol (v/v) (the 1.5%-ethanol group), or 1.0 mL of 5.0% ethanol (v/v) (the 5.0%-ethanol group) (see Materials and methods for details). During the course of the experiment, there were no significant differences among the three groups in terms of the initial and final body weights, as well as the course of body weight changes (Fig 1).

The liver to body weight ratio (g/g, %) and plasma ALT, as well as AST activities, were significantly higher with the higher ethanol dosage at weeks 2 and 10 (Table 1). Plasma and hepatic triglyceride levels were also significantly higher with the higher ethanol dosage at weeks 2 and 10. HE- and oil red O-stained histological images of liver tissues of mice were also examined at week 10 using microscopy. All of these observations consistently indicated liver injury and fatty liver in mice with chronic oral administration of ethanol (S3 Fig for oil red O-staining images).

**Table 1:** Characterization of mice with chronic oral administration of ethanol.

Value^a^	2-wk Group^b^			
	Con	1.5% EtOH	5.0% EtOH	p value
	(*n* = 8)	(*n* = 8)	(*n* = 8)	
Body weight (g)	25.2 ± 1.0	25.3 ± 0.8	24.9 ± 0.6	ns
Liver/body weight ratio	3.66 ± 0.26	3.91 ± 0.21	4.35 ± 0.19	< 0.01
(g/g, %)				
Hepatic triglyceride	23.1 ± 5.0	28.4 ± 2.1	36.6 ± 3.3	< 0.01
(TG (μg)/liver weight (g))				
Plasma AST (U/L)	36.1 ± 5.7	40.5 ± 8.1	52.3 ± 7.9	< 0.01
Plasma ALT (U/L)	24.3 ± 2.7	38.0 ± 6.8	56.8 ± 16.6	< 0.01
Plasma TG (μmol/L)	0.52 ± 0.15	0.63 ± 0.13	0.73 ± 0.15	< 0.05
Value^a^	10-wk Group^b^			
	Con	1.5% EtOH	5.0% EtOH	p value
	(*n* = 8)	(*n* = 8)	(*n* = 7)	
Body weight (g)	26.9 ± 1.5	26.7 ± 0.9	26.3 ± 0.7	ns
Liver/body weight ratio	3.70 ± 0.20	3.89 ± 0.17	4.07 ± 0.18	< 0.01
(g/g, %)				
Hepatic triglyceride	22.1 ± 2.6	26.8 ± 2.1	30.1 ± 2.2	< 0.01
(TG (μg) / liver weight (g))				
Plasma AST (U/L)	33.9 ± 4.5	39.5 ± 2.7	43.4 ± 5.6	< 0.05
Plasma ALT (U/L)	22.6 ± 2.7	27.1 ± 2.3	34.1 ± 4.9	< 0.01
Plasma TG (μmol/L)	0.45 ± 0.05	0.57 ± 0.07	0.65 ± 0.10	< 0.01

^a^ Values of seven to eight independent biological replicates are expressed as the mean ± SD. Data were assessed using ANOVA with Tukey-Kramer test.

^b^ Orally gavaged daily for 2 weeks (2 wk) or 10 weeks (10 wk) with 1.0 mL water (Con), 1.0 mL of 1.5% (v/v) ethanol (1.5% EtOH), or 1.0 mL of 5.0% (v/v) ethanol (5.0% EtOH).

ns, not significant; TG, triglyceride; AST, aspartate aminotransferase; ALT, alanine aminotransferase.

#### Colonic lesions

**Fig 2:**
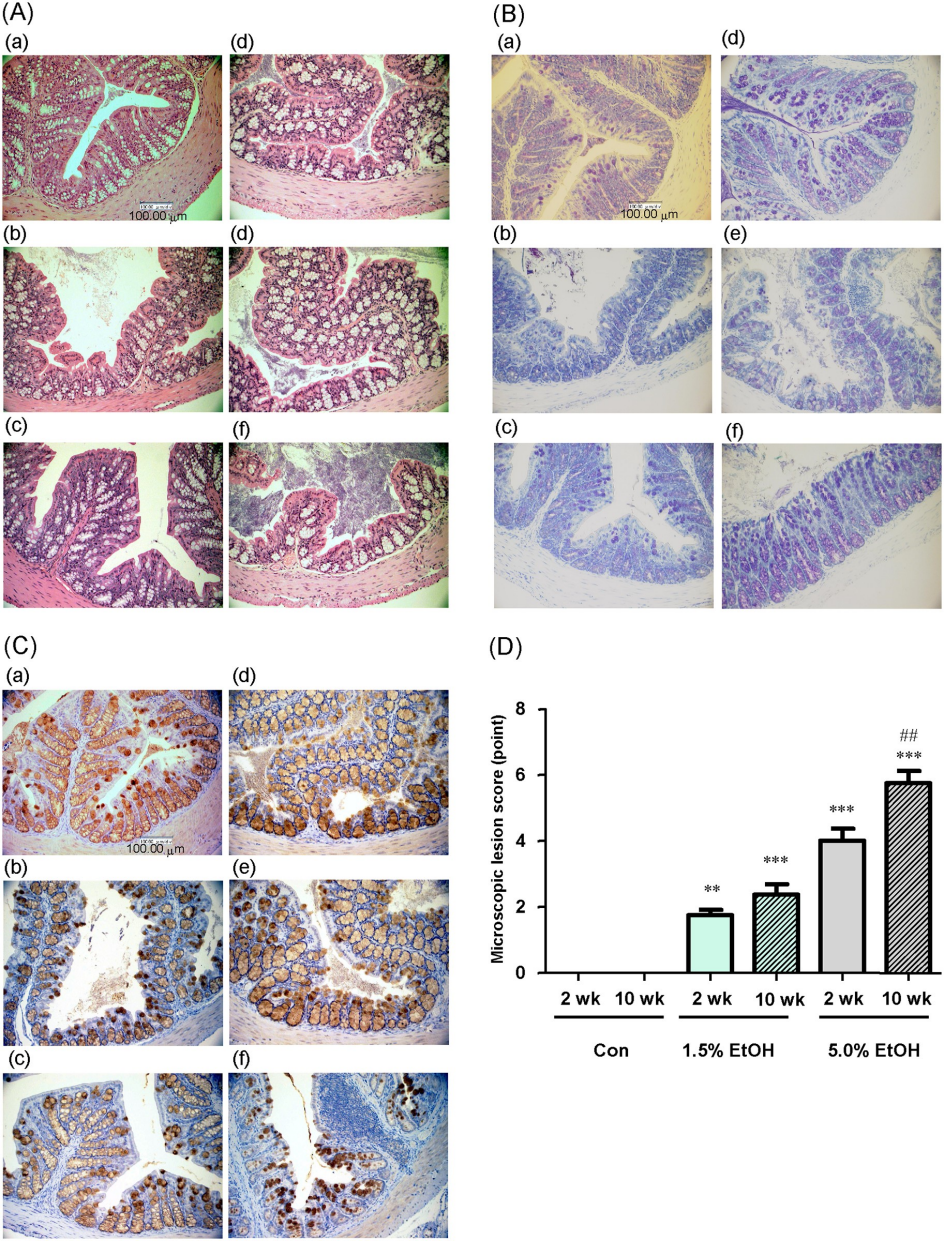
Microscopic observation of colonic mucosa and evaluation of colonic lesions formed with chronic oral administration of ethanol in mice. Eight-week-old mice were orally gavaged daily with 1.0 mL/day of water (Con), 1.0 mL/day of 1.5% (v/v) ethanol (1.5% EtOH), or 1.0 mL/day of 5.0% (v/v) ethanol (5.0% EtOH) for 10 weeks. At weeks 2 and 10 (2 wk and 10 wk, respectively), representative colonic tissue sections were observed microscopically. (A) HE staining images at 400X magnification, (B) TB staining images at 400X magnification, and (C) MUC2 immunohistochemical staining images at 400X magnification. (D) The microscopic lesion scores were evaluated as described in Materials and Methods. The scores are expressed as means ±SD (n = 5). **p<0.01 and ***p<0.001, versus 2 wk Con as assessed by ANOVA with the Tukey-Kramer test. ^##^p<0.01 for 2 wk 5.0% EtOH versus 10 wk 5.0% EtOH as assessed by the paired *t*-test.

The colonic mucosa of the three groups at weeks 2 and 10 was stained with HE (Fig 2A) or TB (Fig 2B) or immunostained using anti-MUC2 antibody (Fig 2C) and observed by microscopy. The colonic mucosa of the mice that received the higher dosage of ethanol for longer periods showed architectural alterations with a thinner mucosal layer (Fig 2A and 2B), which at least in part arose from reduced mucus secretion (Fig 2C), probably due to reduced goblet cell number. The gut tissues (colonic epithelium, as well as colonic crypts, submucosa, and mucosa) of the three groups were also were also evaluated (see Materials and methods for details) by their microscopic lesion scores. The results showed that the lesion scores of colonic tissues (crypts, submucosa, and mucosa) were significantly higher with the higher ethanol dosage and longer periods of ethanol administration (Fig 2D).

#### Effects on colonic oxidative-stress markers

**Fig 3:**
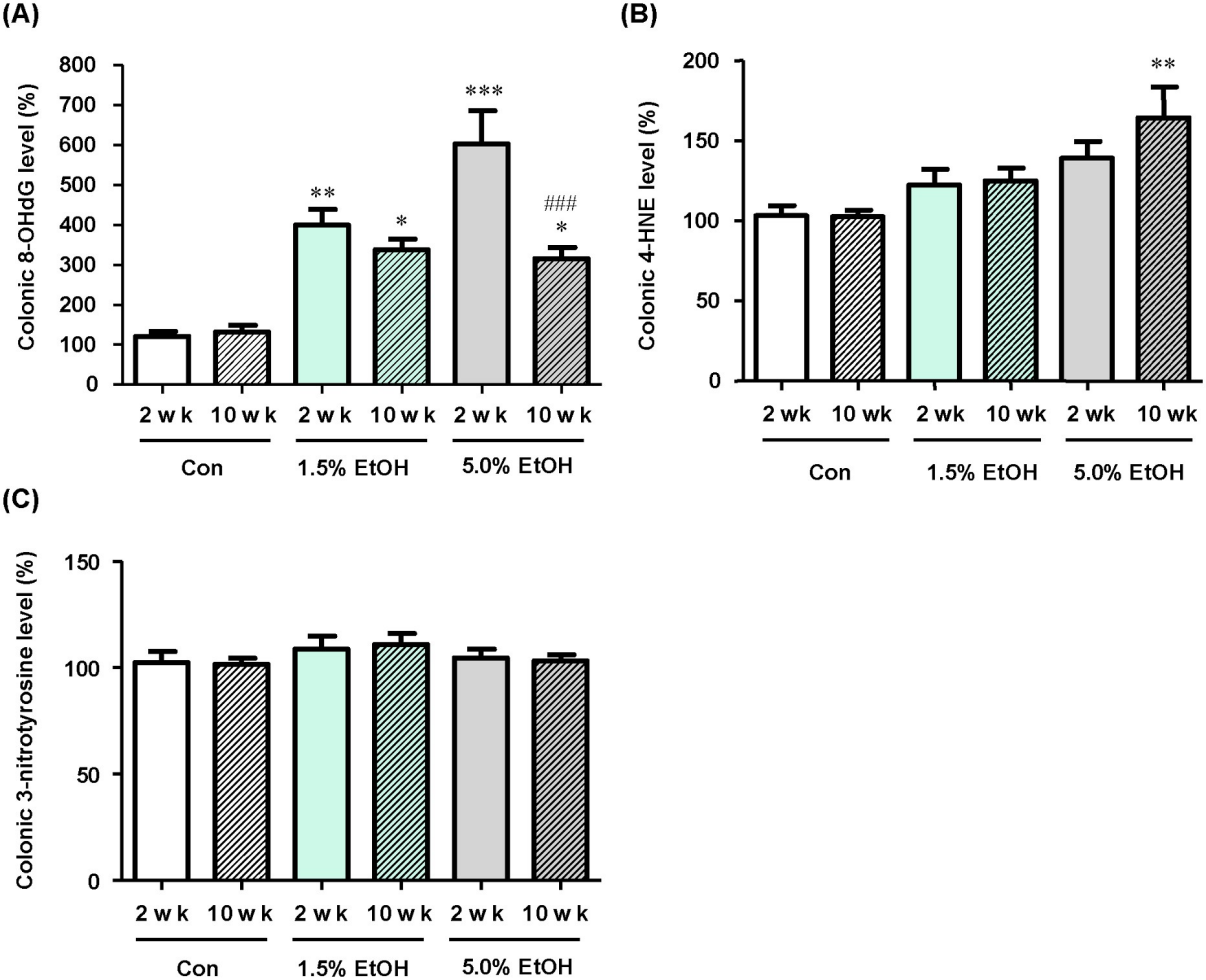
Effects of chronic oral administration of ethanol on the levels of colonic oxidative stress markers in mice. (A) The 8-OHdG levels in colonic tissue extracts were determined by ELISA, and the relative percentages of the 8-OHdG levels are shown with the level obtained with a 2-wk Con subject taken to be 100%. (B) The 4-HNE levels in colonic tissue extracts were determined by ELISA, and the relative percentages of the 4-HNE levels are shown with the level obtained with a 2-wk Con subject taken to be 100%. (C) The nitrotyrosine levels in colonic tissue extracts were determined by ELISA, and the relative percentages of the 3-nitrotyrosine levels are shown with the level obtained with a 2-wk Con subject taken to be 100%. The data are expressed as means ±SD (n = 5). *p<0.05, **p<0.01, and ***p<0.001; versus 2 wk Con as assessed by ANOVA with the Tukey-Kramer test. ^###^p<0.001 for 2 wk 5.0% EtOH versus 10 wk 5.0% EtOH, as assessed by the paired *t*-test. For abbreviations for ethanol-administration conditions, see the legend to Fig 2.

The effects of chronic oral administration of ethanol on the levels of oxidative-stress markers (*i*.*e*., 8-OHdG, 4-HNE, and nitrotyrosine) in the colonic tissues in mice by ELISA (Fig 3A–3C). Immunohistochemical staining with marker-specific antibodies of colonic lamina propria was also examined (S4 Fig). The results were compared among the three groups *(i*.*e*., the 1.5%-ethanol, the 5.0%-ethanol, and the control groups).

At week 2, the 8-OHdG levels were higher with the higher ethanol dosage. At week 10, the 8-OHdG level in the 5.0%-ethanol group was lower than that in the 1.5%-ethanol group, although the levels of both ethanol groups were significantly higher than of the control (Fig 3A). For the 4-HNE levels, the levels appeared to be increased in an ethanol dose- and time-dependent manner (Fig 3B), although only the elevation of the 4HNE level in the 5.0%-ethanol group at week 10 was significant. There were no significant changes in the 3-nitrotyrosine levels during the course of chronic oral administration of ethanol in the three groups (Fig 3C).

### Colonic inflammatory markers

#### Cytokine and chemokine mRNA levels

**Fig 4:**
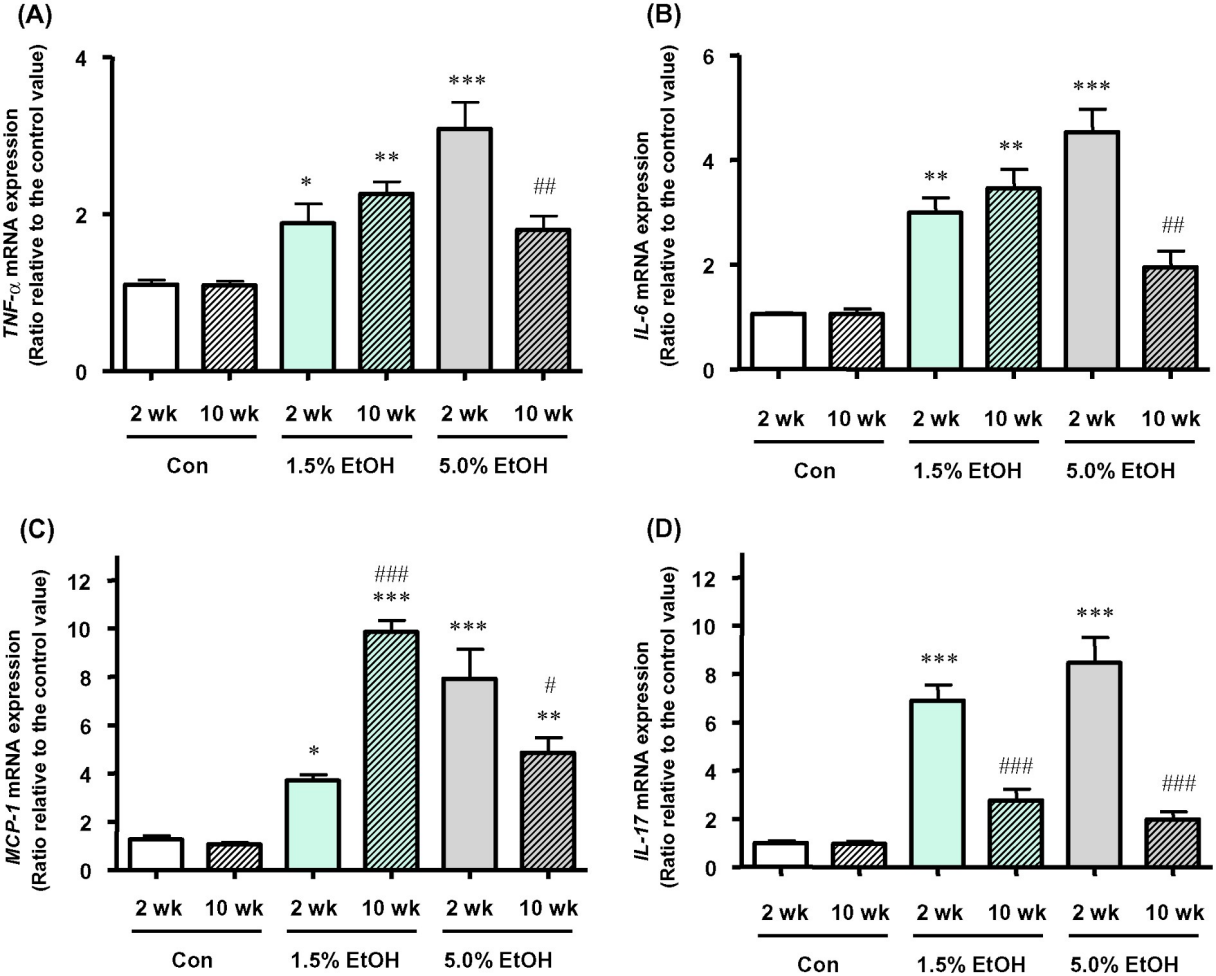
Effects of chronic oral administration of ethanol on colonic mRNA levels of inflammatory marker proteins in mice on qRT-PCR. The mRNA levels of (A) *TNF-α*, (B) *IL-6*, (C) *MCP-1*, and (D) *IL-17A* are shown as relative values with the respective mRNA level of a 2-wk Con subject taken to be 1.0 and expressed as the mean ±SD (n = 5). *p<0.05, **p<0.01, and ***p<0.001; versus 2 wk Con as assessed by ANOVA with the Tukey-Kramer test. ^#^p<0.05, ^##^p<0.01, and ^###^p< 0.001; 2 wk versus 10 wk within the same ethanol dosage, as assessed by the paired *t*-test. For abbreviations for ethanol-administration conditions, see the legend to Fig 2.

The mRNA levels of inflammatory cytokines (TNF-α, IL-6, and IL-17A) and the chemokine MCP-1 in mouse colonic lamina propria were determined by means of qRT-PCR, and the results were compared among the three groups. The expression levels of *TNF-α*, *IL-6*, *IL-17A*, and *MCP-1* mRNAs in colonic tissues were higher with the higher ethanol dosage when the results were compared at week 2 (Fig 4A–4D). For the 1.5%-ethanol group, the expression levels of these mRNAs, except for *IL-17A*, were higher at week 10 than at week 2. For the 5.0%-ethanol group, the expression levels of these four mRNAs at week 10 were lower than those obtained at week 2.

#### Inflammation-associated immune cells

**Fig 5:**
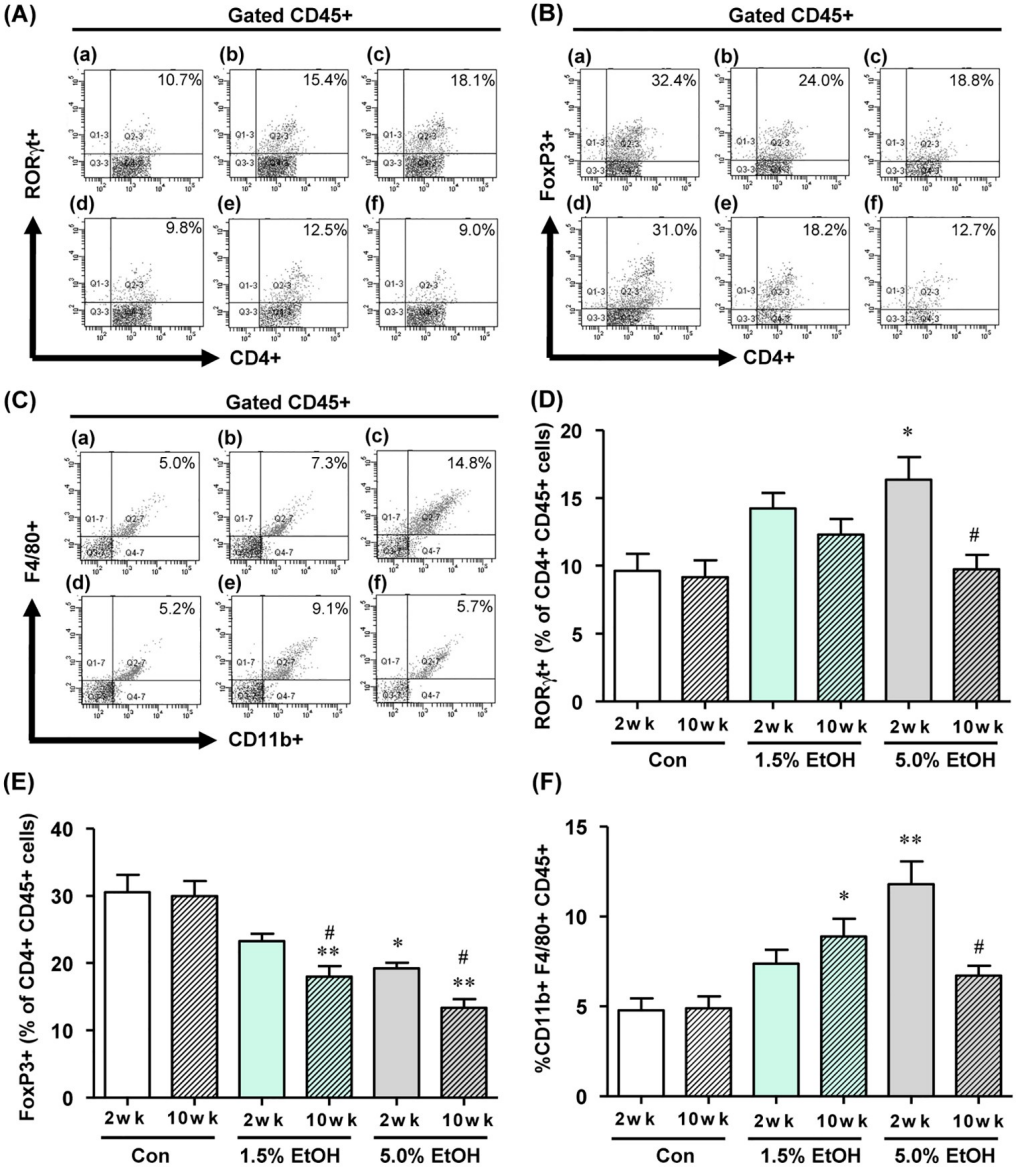
Effects of chronic oral administration of ethanol on the colonic population of inflammation-associated immune cells in mice as analyzed by flow cytometry. (A) The proportion of CD4^+^ CD45^+^ RORγt^+^ T cells, (B) CD4^+^ CD45^+^ Fox3^+^ T cells, (C) CD11b^+^ CD45^+^ F4/80^+^ macrophages detected by flow cytometry: (a) 2-wk Con, (b) 2-wk 1.5% EtOH, (c) 2-wk 5.0% EtOH, (d) 10-wk Con, (e) 10-wk 1.5% EtOH, (f) 10-wk 5.0% EtOH. The quantitative analyses data are shown: (D) CD4^+^ CD45^+^ RORγt^+^ T cells, (E) CD4^+^ CD45^+^ Fox3^+^ T cells, (F) CD11b^+^ CD45^+^ F4/80^+^ macrophages by flow cytometry. For (D), (E) and (F), the results are expressed as means ±SD (n = 4). *p<0.05, **p<0.01 and versus 2 wk control, as assessed by ANOVA with the Tukey-Kramer test. ^#^p<0.05 for 2 wk versus 10 wk with the same ethanol dosage, as assessed by the paired *t*-test. For abbreviations for ethanol-administration conditions, see the legend to Fig 2. For experimental details, see Materials and methods.

The relative abundances of Th17 cells [as CD4^+^ CD45^+^ RORγt-positive (RORγt^+^) cells], Treg cells [as CD4^+^ CD45^+^ Foxp3-positive (FoxP3^+^) cells], and macrophages (as CD11b^+^ CD45^+^ F4/80-positive cells) in colonic lamina propria tissues were analyzed by flow cytometry. (Fig 5A–5F, respectively by flow cytometry analysis; see also S5–S7 Figs for immunofluorescence staining images (RORγt^+^, Foxp3^+^, and F4/80^+^ cells, respectively). The relative abundance of Th17 cells was significantly increased in both ethanol groups at week 2. However, the values were markedly decreased at week 10 in the 5%-ethanol group (Fig 5A and 5D). The relative abundance of Treg cells decreased significantly in an ethanol dose- and time-dependent manner with chronic ethanol administration (Fig 5B and 5E). For the 1.5%-ethanol group, the relative abundance of macrophages increased with increasing periods of chronic ethanol administration; however, for the 5%-ethanol group, the abundance of these cells at week 10 was lower than the value at week 2 (Fig 5C and 5F).

#### AGEs and RAGE levels

**Fig 6:**
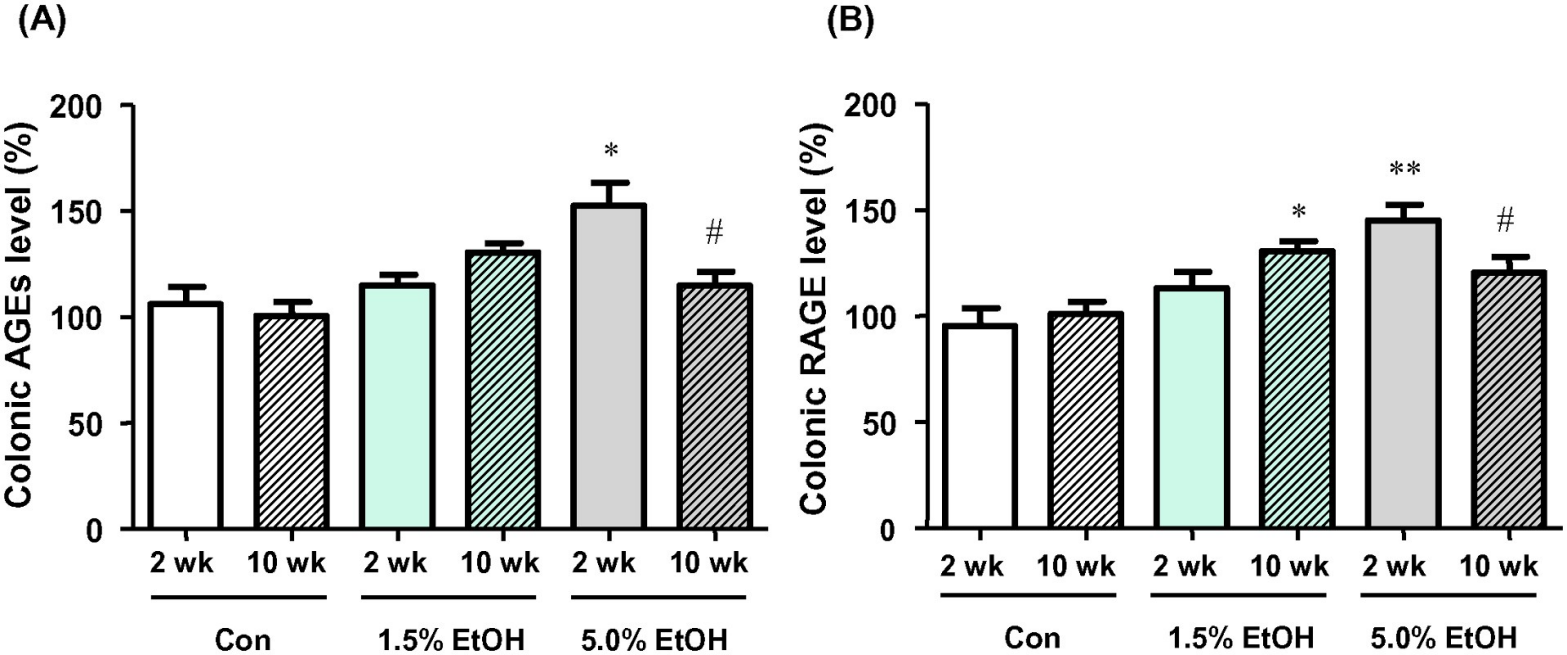
Effects of chronic oral administration of ethanol on AGEs and RAGE levels in colonic tissues in mice. (A) Relative percentages of the AGEs levels, as quantified by ELISA, with the level obtained with a 2-wk Con subject taken to be 100%. (B) Relative percentages of RAGE levels, as quantified by means of ELISA, are shown with the level obtained with a 2-wk Con subject taken to be 100%. Results are means ±SD (n = 5). *p<0.05, **p< 0.01 versus 2 wk Con, as assessed by ANOVA with the Tukey-Kramer test. ^#^p<0.05 for 2 wk versus 10 wk with the same ethanol dosage, as assessed by the paired *t*-test. For abbreviations for ethanol-administration conditions, see the legend to Fig 2.

The relative changes of AGEs and RAGE levels were evaluated by ELISA using the colonic tissue extracts (Fig 6A and 6B; see also S8 Fig for immunohistochemical staining images of colonic lamina propria probed with the AGEs- and the RAGE-specific antibodies). The ELISA results showed that the AGEs and RAGE levels of colonic tissues were elevated with chronic oral administration of ethanol. At week 2, the AGEs levels of the 5.0%-ethanol group were significantly higher than the levels of the other two groups (Fig 6A). However, the AGEs level at week 10 of the 5.0%-ethanol group was lower than the level of the same group at week 2. For the 1.5%-ethanol group, there appeared to be a trend of a time-dependent elevation in the AGEs levels, though this was not significant. Similar observations were obtained on the RAGE levels (Fig 6B). Specifically, at week 2, the RAGE levels of the 5.0%-ethanol group were significantly higher than the levels of other two groups. The RAGE levels at week 10 of the 5.0%-ethanol group were lower than the levels of the same group at week 2. For the 1.5%-ethanol group, there appeared to be a trend for a time-dependent elevation in the RAGE levels (Fig 6B).

#### Gut microbiota structures

**Fig 7:**
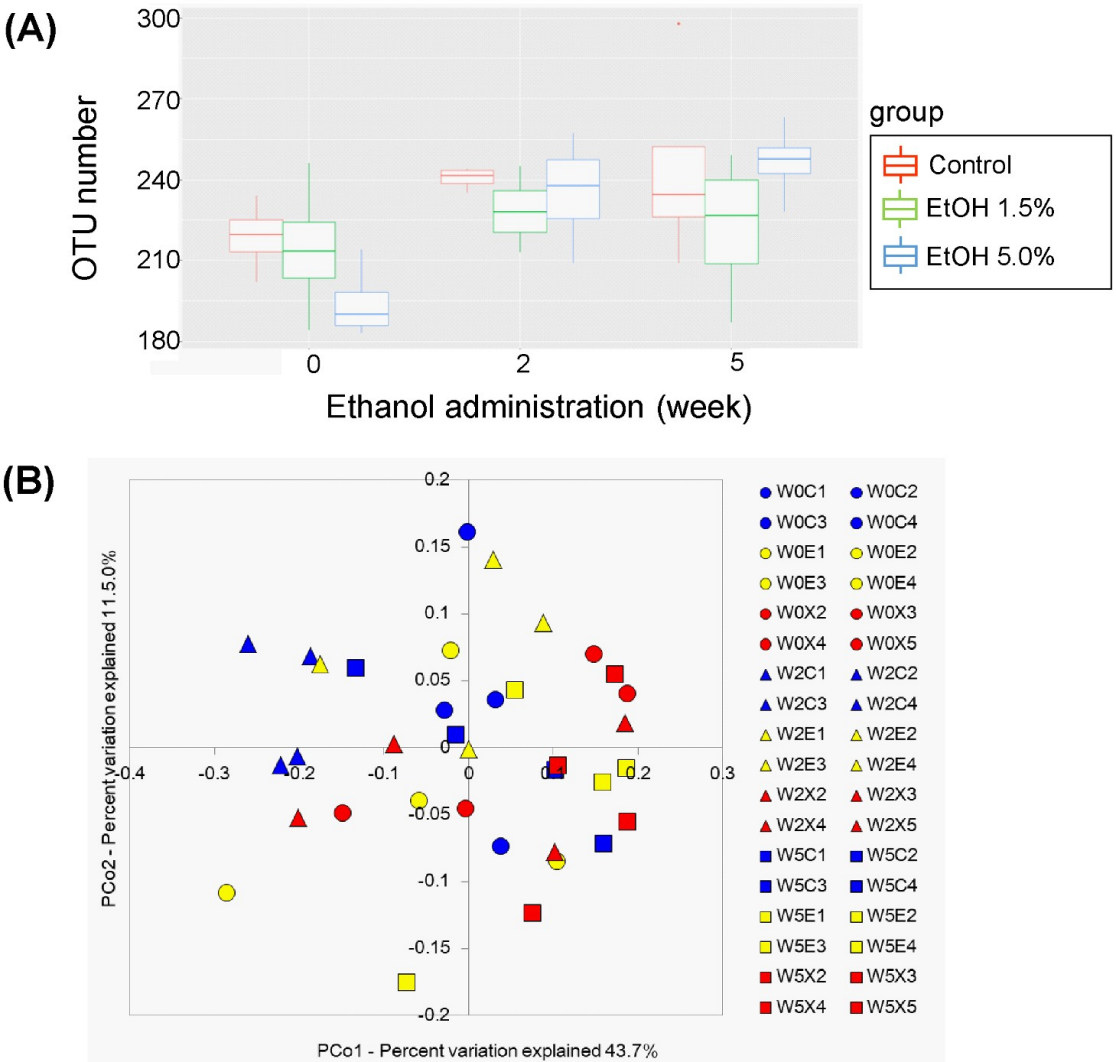
OTU analysis and UniFrac PCoA plots of fecal bacterial communities of the control (Con), 1.5%-ethanol, and 5.0%-ethanol groups. (A) OTU analysis. Box-whisker plots of fecal OTU numbers of the control (red), 1.5%-ethanol (blue), and 5.0%-ethanol (green) groups are shown. (B) UniFrac PCoA plots. The red, blue, and green symbols denote the control (Con), the 1.5%-ethanol, and the 5.0%-ethanol groups, respectively. Circle, triangle, and square symbols denote 0 weeks, 2 weeks, and 5 weeks, respectively, of chronic ethanol administration. For subject names, “W0C1”, for example, refers to mouse #1 of control group (C) at week 0 (W0), “W2E3” refers to mouse #3 of the 1.5% ethanol group (E) at week 2 (W2), and “W5X4” refers to mouse #4 of the 5.0% ethanol group (X) at week 5 (W5).

**Fig 8:**
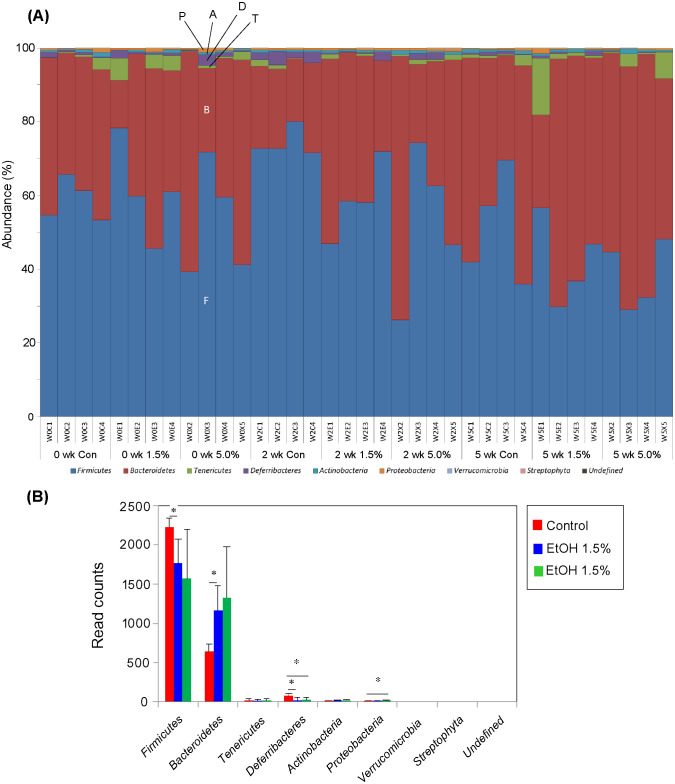
Relative abundances of bacterial phyla in the fecal bacterial communities of the three groups. (A) Relative abundances of bacterial phyla are compared among individual subjects. For subject names, see the legend to Fig 8 (B). Phyla shown are, from bottom to top, *Firmicutes* (F, blue), *Bacteroidetes* (B, brown), *Tenericutes* (T, green), *Defferibacteres* (D, purple), *Actinobacteria* (A, sky blue), *Proteobacteria* (P, orange), *Verrucomicrobia* (light blue), *Streptophyta* (pink), and unidentified (black). For convenience sake, the six top phyla are indicated with one-letter notation on the W0X3 bar. (B) Relative abundances of bacterial phyla are compared among at week 5 **c**ontrol (red), 1.5%-ethanol (blue), and 5.0%-ethanol (green) groups. Error bars indicate ±SD (n = 4) within the group. *p<0.05 versus control, as assessed by Welch’s *t*-test.

The microbiotas of the feces obtained from the three groups (4 mice/group) were characterized at week 0, 2, and 5 and compared with each other (fecal microbiotas at week 10 could not be analyzed). The fecal species richness (α-diversity) of these groups was assessed by comparing the number of species-level OTUs among the individuals, defined as the number of clusters sharing ≥ 96% sequence identity. No significant difference in the OTU numbers could be found among these three groups (Fig 7A). Phylogenetic diversity was then analyzed among the fecal microbiota structures (*i*.*e*., β-diversity) using multivariate methods (namely, principal coordinate analysis; PCoA). The UniFrac-PCoA analyses showed no appreciable clustering of the gut microbiota structures in each group (Fig 7B). The fecal mictobiota phylogenies were then compared among the three groups at the phylum level. In order of decreasing abundance, the common phyla in the fecal microbiota of control mice were *Firmicutes*, *Bacteroidetes*, and *Deferribacteres* (Fig 8A). Although the same phyla were found at week 5 in feces of the 1.5%-ethanol and 5.0%-ethanol groups, *Firmicutes* formed a significantly smaller proportion in the 1.5%-ethanol group than in control mice, whereas *Deferribacteres* formed a significantly smaller proportion in the 1.5%-ethanol and 5.0%-ethanol groups than in control mice (Fig 8B). The relative abundances of *Bacteroidetes* and *Proteobacteria* were significantly increased at week 5 feces of 1.5%-ethanol and the 5.0%-ethanol groups, respectively.

## Discussion

Ethanol is known to induce ROS production and tissue inflammation via a variety of cellular processes, which are intrinsically linked to carcinogenesis [2, 5, 6]. Our previous results consistently showed that the gut microbiotas of alcoholic patients, a high-risk group for ER-CRC, were diminished in dominant obligate anaerobes, such as *Bacteroides*, and enriched in streptococci and other minor facultative anaerobes, consistent with the proposed chronic ROS formation in the gut of these patients [12]. The present study confirmed that chronic oral administration of ethanol indeed resulted in the elevation of the levels of oxidative stress markers (8-OHdG and 4HNE) from the control level in the colonic tissues in mice (Fig 3). Moreover, the mRNA levels of colonic inflammatory cytokines (TNF-α, IL-6, and IL-17A) and the chemokine MCP-1 were all elevated from the control level with chronic oral administration of ethanol (Fig 4A–4D), and the relative abundances of Th17 cells and macrophages in the colonic tissues were greater in the ethanol groups than in the control (Fig 5D and 5F). The relative abundances of Treg cells [21] decreased in an ethanol dosage- and time-dependent manner (Fig 5E). All of these observations were consistent with the suggested enhancement of inflammation of colonic tissues in the ethanol groups. This could also consistently account for the fact that the colonic tissues of mice that received a higher dosage of ethanol for longer periods showed a thinner mucosal layer and higher lesion scores (Fig 2), because colorectal lesions tend to be associated with inflammation [22]. However, chronic long-term (*i*.*e*., for 10 weeks) administration of a high dosage (*i*.e, 5.0% ethanol (v/v)) of ethanol lowered the levels of a colonic oxidative stress marker (8-OHdG, see Fig 3A) and colonic inflammatory cytokines (Fig 4), as well as the relative abundances of colonic mature Th17 cells (CD4^+^ CD45^+^ RORγt^+^) (Fig 5D) and macrophages (Fig 5F), when these levels were compared with those observed with a shorter-term (*i*.*e*., for 2 weeks) or a lower dosage (*i*.*e*., 1.5% ethanol (v/v)) of chronic ethanol administration. These observations appear to be linked to the observed significant decrease in the relative abundances of colonic mature Treg cells (CD4^+^ CD45^+^ FoxP3^+^) with short-term 5.0% ethanol (v/v) and chronic long-term 1.5% ethanol (v/v) administration (Fig 5E). It would be likely that chronic long-term administration of an excess amount of ethanol might eventually undermine the gut tissues and immune system functions in mice.

The colonic microbiota of the ethanol groups were found to be enriched in *Bacteroidetes* and diminished in *Firmicutes* and *Deferribacters*, and that of the 5.0%-ethanol group was enriched in *Proteobacteria* and diminished in *Deferribacters*. These alterations of microbiota structures with chronic oral administration of ethanol were consistent with those previously observed with mice administered 5.0% ethanol [23]. It must be mentioned that these alterations were reminiscent of human inflammatory bowel disease [24], which was enriched in *Bacteroidetes*, rather than those observed in alcoholic patients (see above and [12]). Changes of human gut microbiota structure to the alcoholic structure likely occur after many years (even more than a decade) of habitual drinking and might be difficult to reproduce using mouse models during the examined periods (*i*.*e*., for 10 weeks) of ethanol administration, which likely resulted in an acute inflammatory state of the colonic tissues (see above). The inability to precisely reproduce the microbiological consequences of alcoholism using a mouse model may be in part due to a large longevity gap between mice and humans and could be a limitation of this study.

In the present study, chronic oral administration of ethanol resulted in the elevation of the levels of AGEs and RAGE in the colonic tissues in mice. Although the detailed mechanism of the observed elevation of AGEs levels remains to be clarified, the formation of AGEs is known to be initiated by the formation of highly reactive α-dicarbonyls produced via different pathways [13], some of which could potentially arise from chronic ethanol consumption. For example, ethanol-induced ROS potentially triggers the Namiki and Wolff pathways of α-dicarbonyls formation [25, 26]. In addition, the observed enhancement of the 4-HNE levels in the colonic tissues with chronic ethanol administration (see Results) suggests the formation of lipid peroxides in the colon, which also serves as a source of reactive carbonyl species responsible for AGEs formation [27]. Moreover, catabolism of ethanol-induced ketone bodies could also give rise to the reactive carbonyl species [28]. Additionally, the ingestion of ethanol results in elevated blood acetaldehyde (AcH) levels, which may lead to the formation of acetaldehyde-derived AGEs (AcH-AGEs) [29].

On binding of AGEs to RAGE, multiple signaling pathways are activated to cause multiple pathological effects associated with oxidative stress and inflammation [13, 14]. For example, binding of AGEs to RAGE activates NADPH oxidase, which catalyzes the production of ROS to result in an enhancement of cellular oxidative stress [30]. It also activates transcription of nuclear factor κB (NF-κB), a transcription factor involved in different signaling pathways including transcription activation of pro-inflammatory cytokines (*i*.*e*., TNF-α and IL-6) [31, 32]. TNF-α induces expression of chemokines (*i*.*e*., MCP-1) from a variety of leukocytes, thereby having an indirect migration effect on macrophages [33]. It has been shown that ROS also activates NF-κB [30], and NF-κB is capable of binding to RAGE [34, 35]. These contribute to an establishment of a positive feedback cycle of NF-κB expression, which results in a shift from a pro-inflammatory state to a chronic pathophysiological state [14, 34, 35]. Thus, RAGE-mediated signaling has been implicated as a link between the accumulation of AGEs and development (*e*.*g*., initiation, progression, and metastasis) of many types of cancers by triggering oxidative stress and chronic inflammatory responses in both humans and mice [14]. The results of the present ELISA analysis showed elevated levels of RAGE in the colonic tissues of mice of the 1.5%-ethanol group (at week 10) and of the 5.0%-ethanol group (at week 2). The enhanced RAGE-mediated signaling pathway could exacerbate the oxidative stress and the ethanol-induced inflammatory state of colonic tissues and at least partly contribute to ER-CRC pathogenesis. Further studies are needed to clarify the detailed mechanism of ethanol-induced elevation of the AGEs and RAGE levels and the roles of these molecules in ER-CRC pathogenesis.

## Conclusions

In this study, elevations of oxidative stress and inflammatory markers in mice colonic tissues were seen with chronic ethanol administration. Alteration of fecal microbiota structures of mice with chronic ethanol administration was reminiscent of those observed in human inflammatory bowel disease, which was enriched in *Bacteroidetes*. Furthermore, chronic ethanol administration was found to result in elevated levels of AGEs and RAGE in the mice colonic tissues, implying the possibility that ethanol-induced RAGE signaling exacerbates inflammation in the colon and partly contribute to the pathogenesis of ER-CRC.

## Supporting information

S1 File ARRIVE guidelines 2.0 author checklist. (PDF)

S1 Table Nucleotide sequences of primers used for qRT-PCR. (TIF)

S1 Fig Experiment design. (TIF)

S2 Fig Gating strategies for fluorescence activated cell sorting. Flow cytometry panels of gating processes for isolation of CD4^+^ cells [panels (A) through (E)] and CD11b^+^ cells [panels (G) through (K)] are shown with their hierarchical strategies [panels (F) and (L), respectively].
(TIF)

S3 Fig Effects of chronic oral administration of ethanol on the levels of fatty liver in mice on oil red-O staining. Oil-red O staining of liver sections (at 1,000X magnification) shows that alcohol induces an increase in hepatic triglyceride levels in both a dose-dependent and a time-dependent manner.
(TIF)

S4 Fig Effects of chronic oral administration of ethanol on the levels of colonic oxidative-stress markers in mice on immunohistochemical staining. Images of colonic lamina propria after immunohistochemical staining using (A) monoclonal anti-8-OHdG antibody, (B) monoclonal anti-4-HNE antibody and (C) monoclonal anti-nitrotyrosine antibody are shown at 400X magnification.
(TIF)

S5 Fig Effects of chronic oral administration of ethanol on the colonic population of RORγt-positive cells in mice on immunofluorescence staining. Images of colonic lamina propria after immunofluorescence staining using anti-CD4 antibody (green), anti-RORγt antibody (red), DAPI (blue) and combined fluorescence (merge) are observed with a fluorescence microscope. (a) 2-wk control, (b) 2-wk 1.5% EtOH, (c) 2-wk 5.0% EtOH, (d) 10-wk control, (e) 10-wk 1.5% EtOH, and (f) 10-wk 5.0% EtOH.
(TIF)

S6 Fig Effects of chronic oral administration of ethanol on the colonic population of Fox3-positive cells in mice on immunofluorescence staining. Images of colonic lamina propria after immunofluorescence staining using anti-CD4 antibody (green), anti-Foxp3 antibody (red), DAPI (blue) and combined fluorescence (merge) are observed with a fluorescence microscope. (a) 2-wk control, (b) 2-wk 1.5% EtOH, (c) 2-wk 5.0% EtOH, (d) 10-wk control, (e) 10-wk 1.5% EtOH, (f) 10-wk 5.0% EtOH.
(TIF)

S7 Fig Effects of chronic oral administration of ethanol on the colonic population of F4/80-positive cells in mice on immunofluorescence staining. Images of colonic lamina propria after immunofluorescence staining using anti-iNOS antibody (green), anti-F4/80 antibody (red), DAPI (blue) and combined fluorescence (merge) are observed with a fluorescence microscope. (a) 2-wk control, (b) 2-wk 1.5% EtOH, (c) 2-wk 5.0% EtOH, (d) 10-wk control, (e) 10-wk 1.5% EtOH, (f) 10-wk 5.0% EtOH.
(TIF)

S8 Fig Effects of chronic oral administration of ethanol on AGE and RAGE levels in colonic tissues in mice on immunohistochemical staining. Images of colonic lamina propria after immunohistochemical staining using (A) polyclonal anti-AGEs antibody and (B) polyclonal anti-RAGE antibody are shown at 400X magnification. For abbreviations for ethanol-administration conditions, see the legend to Fig 2. For experimental details, see Materials and methods.
(TIF)

## References

[1] Mizoue T, Inoue M, Wakai K, Nagata C, Shimazu T, Tsuji I, et al Alcohol drinking and colorectal cancer in Japanese: a pooled analysis of results from five cohort studies. Am J Epidemiol. 2008;167(12):1397–1406. 10.1093/aje/kwn073 18420544

[2] Seitz HK, Stickel F. Molecular mechanisms of alcohol-mediated carcinogenesis. Nat Rev Cancer. 2007;7(8):599–612. 10.1038/nrc2191 17646865

[3] Mizukami T, Yokoyama A, Yokoyama T, Onuki S, Maruyama K. Screening by total colonoscopy following fecal immunochemical tests and determinants of colorectal neoplasia in Japanese men with alcohol dependence. Alcohol Alcohol. 2017;52(2):131–137. 10.1093/alcalc/agw071 28182201

[4] Halsted CH, Robles EA, Mezey E. Distribution of ethanol in the human gastrointestinal tract. Am J Clin Nutr. 1973;26(8):831–834. 10.1093/ajcn/26.8.831 4720670

[5] Na HK, Lee JY. Molecular basis of alcohol-related gastric and colon cancer. Int J Mol Sci. 2017;18(6):1116 10.3390/ijms18061116 28538665 PMC5485940

[6] Rossi M, Jahanzaib Anwar M, Usman A, Keshavarzian A, Bishehsari F. Colorectal Cancer and Alcohol Consumption-Populations to Molecules. Cancers (Basel). 2018;10(2):E38 10.3390/cancers10020038 29385712 PMC5836070

[7] Das SK, Vasudevan DM. Alcohol-induced oxidative stress. Life Sci. 2007;81(3):177–187. 10.1016/j.lfs.2007.05.005 17570440

[8] Wu D, Cederbaum AI. Alcohol, oxidative stress, and free radical damage. Alcohol Res Health. 2003;27(4):277–284. 15540798 PMC6668865

[9] Linhart K, Bartsch H, Seitz HK. The role of reactive oxygen species (ROS) and cytochrome P-450 2E1 in the generation of carcinogenic ethano-DNA adducts. Redox Biol. 2014;3:56–62. 10.1016/j.redox.2014.08.009 25462066 PMC4297928

[10] Simanowski UA, Suter P, Russell RM, Heller M, Waldherr R, Ward R, et al Enhancement of ethanol induced rectal mucosal hyper regeneration with age in F344 rats. Gut. 1994;35(8):1102–1106. 10.1136/gut.35.8.1102 7926914 PMC1375063

[11] Purohit V, Gao B, Song BJ. Molecular mechanisms of alcoholic fatty liver Alcoholism. Clinical and Experimental Research. 2009;33(2):191–205. 10.1111/j.1530-0277.2008.00827.x PMC2633431 19032584

[12] Tsuruya A, Kuwahara A, Saito Y, Yamaguchi H, Tsubo T, Suga S, et al Ecophysiological consequences of alcoholism on human gutmicrobiota: implications for ethanol-related pathogenesis of colon cancer. Sci. Rep. 2016;6: 27923 10.1038/srep27923 27295340 PMC4904738

[13] Ott C, Jacobs K, Haucke E, Navarrete Santos A, Grune T, Simm A. Role of advanced glycation end products in cellular signaling. Redox Biol. 2014;2:411–429. 10.1016/j.redox.2013.12.016 24624331 PMC3949097

[14] Schröter D, Höhn A. Role of advanced glycation end products in carcinogenesis and their therapeutic implications. Current Pharmaceutical Design. 2018;24(44):5245–5251. 10.2174/1381612825666190130145549 30706806 PMC6635609

[15] Sakellariou S, Fragkou P, Levidou G, Gargalionis AN, Piperi C, Dalagiorgou G, et al Clinical significance of AGE-RAGE axis in colorectal cancer: associations with glyoxalase-I, adiponectin receptor expression and prognosis. BMC Cancer. 2016;16:174 10.1186/s12885-016-2213-5 26931562 PMC4774155

[16] Moura FA, Goulart MOF, Campos SBG, da Paz Martins AS. The Close Interplay of Nitro-Oxidative Stress, Advanced Glycation end Products and Inflammation in Inflammatory Bowel Diseases. Curr Med Chem. 2020: 27(13): 2059–2076. 10.2174/0929867325666180904115633 30182837

[17] Piperi C, Adamopoulos C, Papavassiliou AG. Potential of glycative stress targeting for cancer prevention. Cancer Lett. 2017: 390: 153–159. 10.1016/j.canlet.2017.01.020 28111136

[18] Shih MF, Taberner PV. Dose-dependent effects of chronic ethanol on mouse adipose tissue lipase activity and cyclic AMP accumulation. Br J Pharmacol. 1997: 120(4): 721–7. 10.1038/sj.bjp.0700973 9051314 PMC1564519

[19] Appleyard CB, Wallace JL. Reactivation of hapten-induced colitis and its prevention by anti-inflammatory drugs. Am J Physiol. 1995;269:G119–125. 10.1152/ajpgi.1995.269.1.G119 7631788

[20] Tahan G, Gramignoli R, Marongiu F, Aktolga S, Cetinkaya A, Tahan V, et al Melatonin expresses powerful anti-inflammatory and antioxidant activities resulting in complete improvement of acetic-acid-induced colitis in rats. Dig Dis Sci. 2011;56(3):715–720. 10.1007/s10620-010-1364-5 20676767

[21] Barbi J, Pardoll D, Pan F. Treg functional stability and its responsiveness to the microenvironment. Immunol Rev. 2014;259(1):115–139. 10.1111/imr.12172 24712463 PMC3996455

[22] Lord R, Burr NE, Mohammed N, Subramanian V. Colonic lesion characterization in inflammatory bowel disease: A systematic review and meta-analysis. World J Gastroenterol. 2018;24(10):1167–1180. 10.3748/wjg.v24.i10.1167 29563760 PMC5850135

[23] Bull-Otterson L, Feng W, Kirpich I, Wang Y, Qin X, Liu Y, et al Metagenomic analyses of alcohol induced pathogenic alterations in the intestinal microbiome and the effect of Lactobacillus rhamnosus GG treatment. PloS one. 2013;8(1):e53028 10.1371/journal.pone.0053028 23326376 PMC3541399

[24] Manichanh C, Borruel N, Casellas F, Guarner F. The gut microbiota in IBD. Nat Rev Gastroenterol Hepatol. 2012;9(10):599–608. 10.1038/nrgastro.2012.152 22907164

[25] Namiki M. Chemistry of Maillard reactions: recent studies on the browning reaction mechanism and the development of antioxidants and mutagens. Adv Food Res. 1988;32:115–184. 10.1016/s0065-2628(08)60287-6 3075879

[26] Wolff SP, Dean RT. Glucose autoxidation and protein modification. The potential role of ‘autoxidative glycosylation’ in diabetes. Biochem J. 1987;245(1):243–250. 10.1042/bj2450243 3117042 PMC1148106

[27] Bucala R, Makita Z, Koschinsky T, Cerami A, Vlassara H. Lipid advanced glycosylation: pathway for lipid oxidation in vivo. Proc Natl Acad Sci USA. 1993;90(14):6434–6438. 10.1073/pnas.90.14.6434 8341651 PMC46946

[28] Thornalley PJ. Pharmacology of methylglyoxal: formation, modification of proteins and nucleic acids, and enzymatic detoxification—a role in pathogenesis and antiproliferative chemotherapy. Gen Pharmacol. 1996;27(4):565–573. 10.1016/0306-3623(95)02054-3 8853285

[29] Hayashi N, George J, Takeuchi M, Fukumura A, Toshikuni N, Arisawa T, et al Acetaldehyde-derived advanced glycation end-products promote alcoholic liver disease. PLoS ONE. 2013;8(7):e70034 10.1371/journal.pone.0070034 23922897 PMC3724722

[30] Wautier MP, Chappey O, Corda S, Stern DM, Schmidt AM, Wautier JL. Activation of NADPH oxidase by AGE links oxidant stress to altered gene expression via RAGE. Am J Physiol Endocrinol Metab. 2001;280(5):E685–694. 10.1152/ajpendo.2001.280.5.E685 11287350

[31] Yan SD, Schmidt AM, Anderson GM, Zhang J, Brett J, Zou YS, et al Enhanced cellular oxidant stress by the interaction of advanced glycation end products with their receptors/binding proteins. J Biol Chem. 1994;269(13):9889–9897. 8144582

[32] Brownlee M. Advanced protein glycosylation in diabetes and aging. Annu Rev Med. 1995;46:223–234. 10.1146/annurev.med.46.1.223 7598459

[33] Danese S, Mantovani A. Inflammatory bowel disease and intestinal cancer: a paradigm of the Yin-Yang interplay between inflammation and cancer. Oncogene. 2010;29(23):3313–3323. 10.1038/onc.2010.109 20400974

[34] Lin L, Park S, Lakatta EG. RAGE signaling in inflammation and arterial aging. Front Biosci. 2009;14:1403–1413. 10.2741/3315 19273137 PMC2661616

[35] Andrassy M, Igwe J, Autschbach F, Volz C, Remppis A, Neurath MF, et al Posttranslationally modified proteins as mediators of sustained intestinal inflammation. Am J Pathol. 2006;169(4):1223–1237. 10.2353/ajpath.2006.050713 17003481 PMC1780182

