# Supplementary figures and images for: Alteration of oxidative-stress and related marker levels in mouse colonic tissues and fecal microbiota structures with chronic ethanol administration: Implications for the pathogenesis of ethanol-related colorectal cancer
Source: PLoS One. 2021 Feb 12;16(2):e0246580. doi: 10.1371/journal.pone.0246580 (PMC7880462; doi:10.1371/journal.pone.0246580)

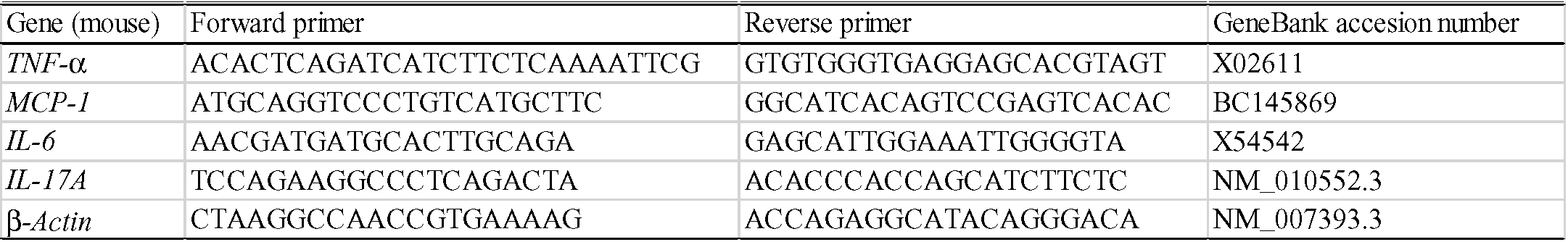

Supplement: S1 Table — (TIF) [file pone.0246580.s002.tif]

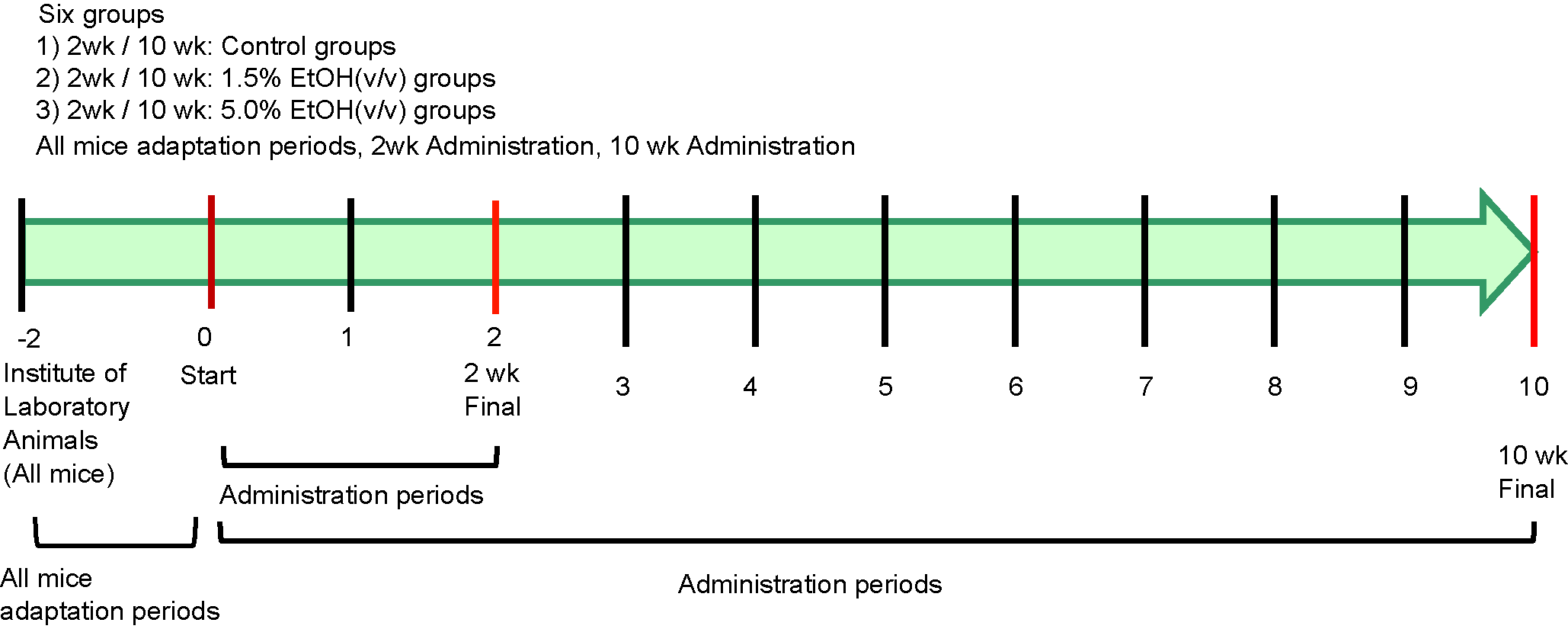

Supplement: S1 Fig — (TIF) [file pone.0246580.s003.tif]

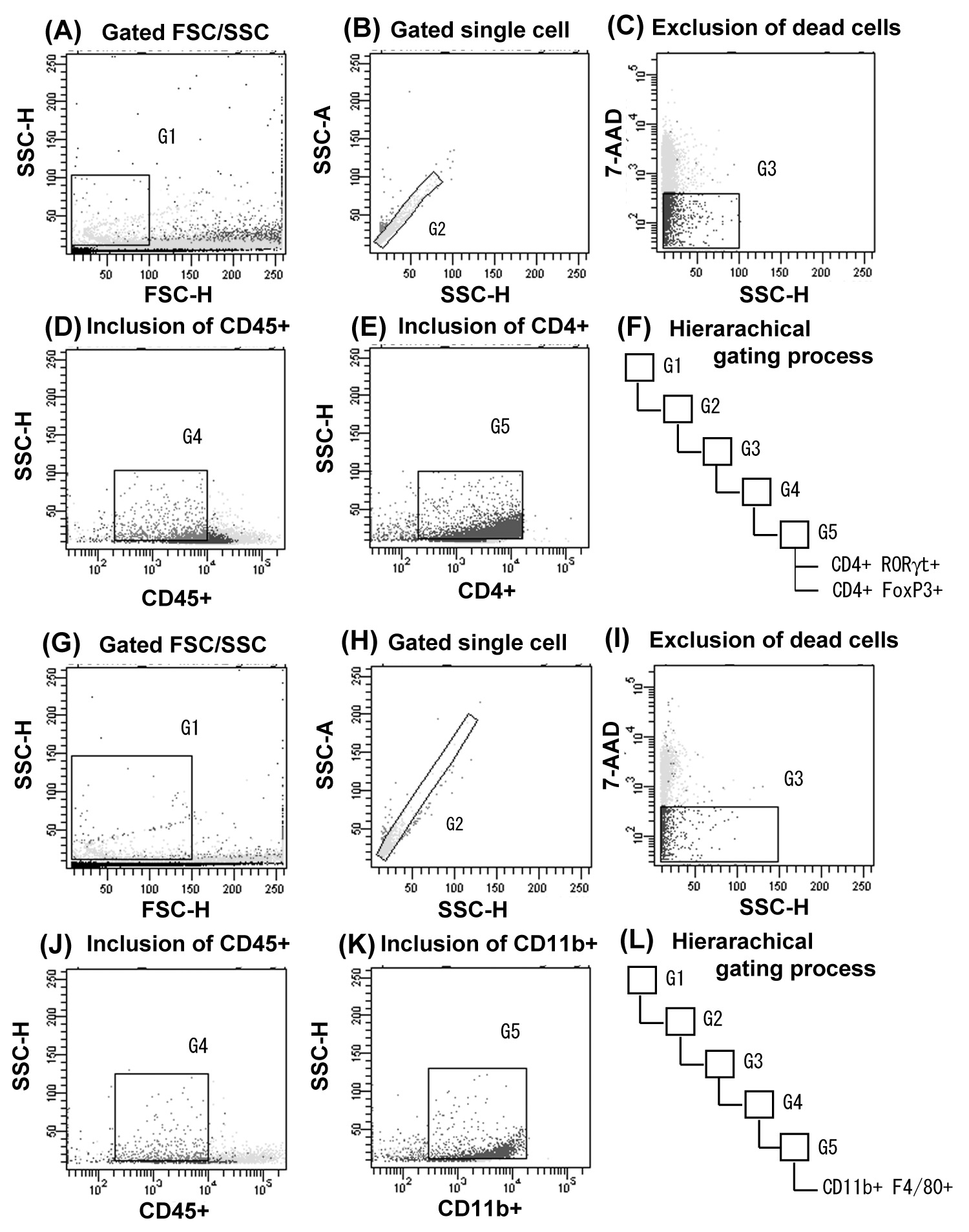

Supplement: S2 Fig — Flow cytometry panels of gating processes for isolation of CD4+ cells [panels (A) through (E)] and CD11b+ cells [panels (G) through (K)] are shown with their hierarchical strategies [panels (F) and (L), respectively]. (TIF) [file pone.0246580.s004.tif]

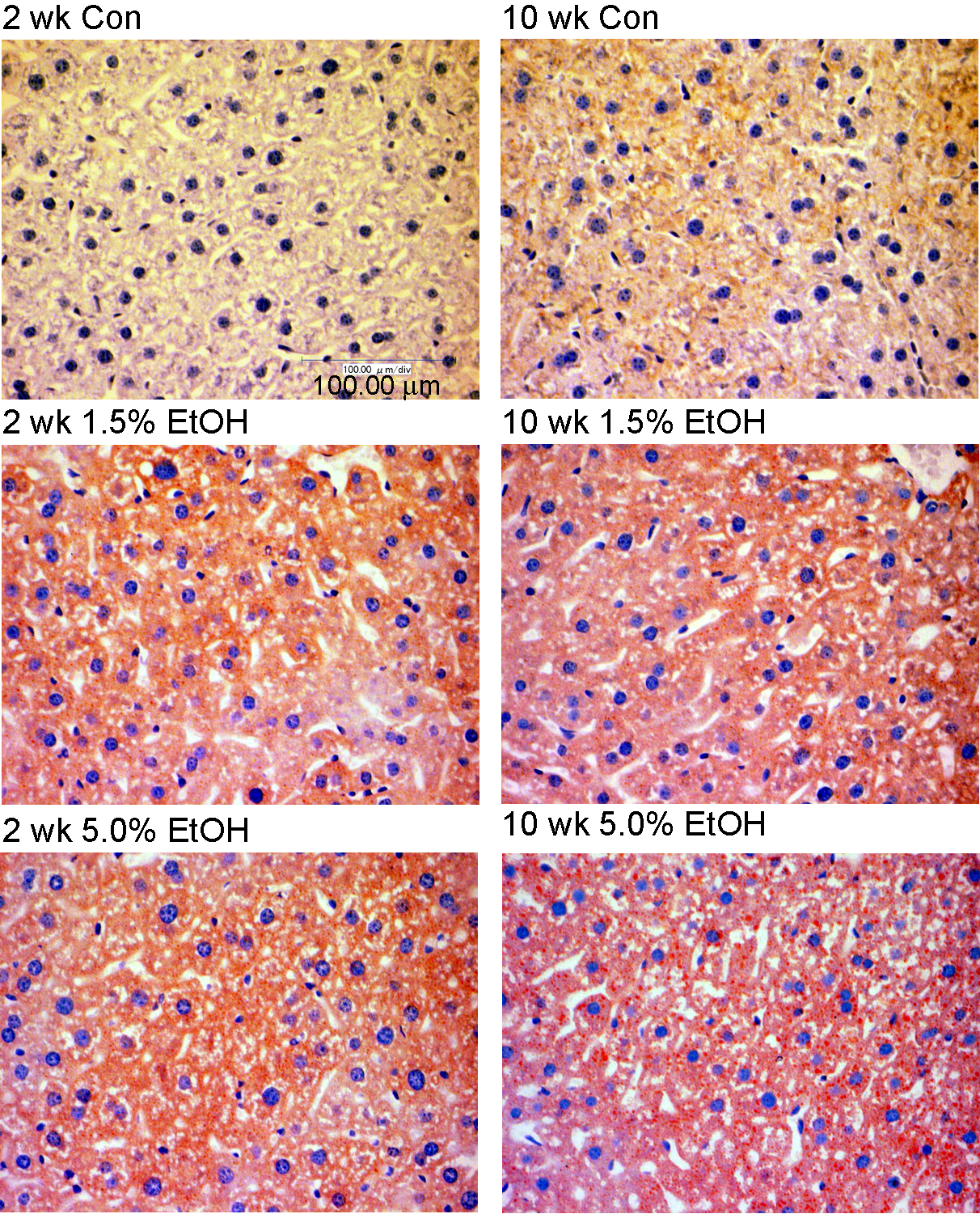

Supplement: S3 Fig — Oil-red O staining of liver sections (at 1,000X magnification) shows that alcohol induces an increase in hepatic triglyceride levels in both a dose-dependent and a time-dependent manner. (TIF) [file pone.0246580.s005.tif]

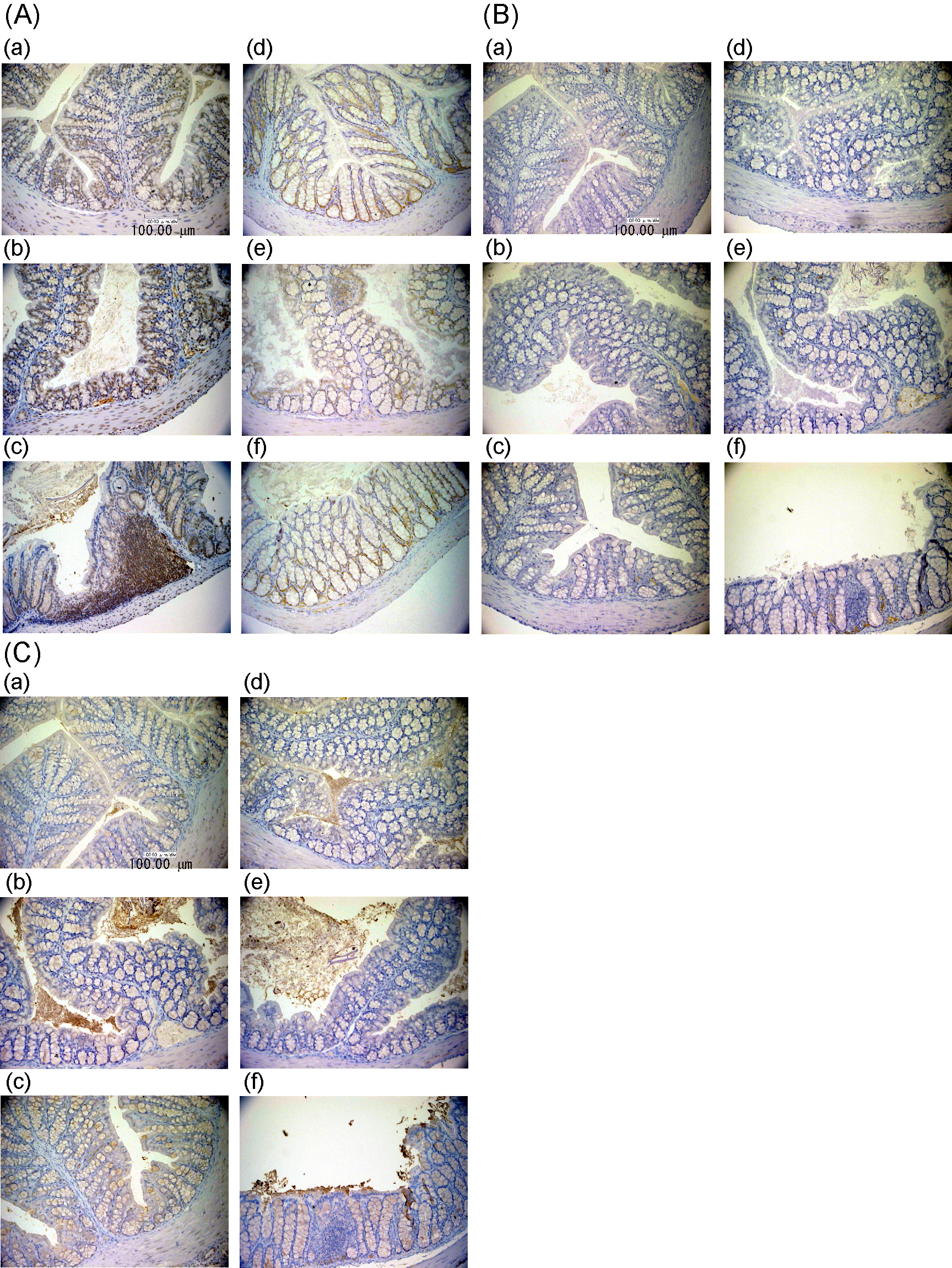

Supplement: S4 Fig — Images of colonic lamina propria after immunohistochemical staining using (A) monoclonal anti-8-OHdG antibody, (B) monoclonal anti-4-HNE antibody and (C) monoclonal anti-nitrotyrosine antibody are shown at 400X magnification. (TIF) [file pone.0246580.s006.tif]

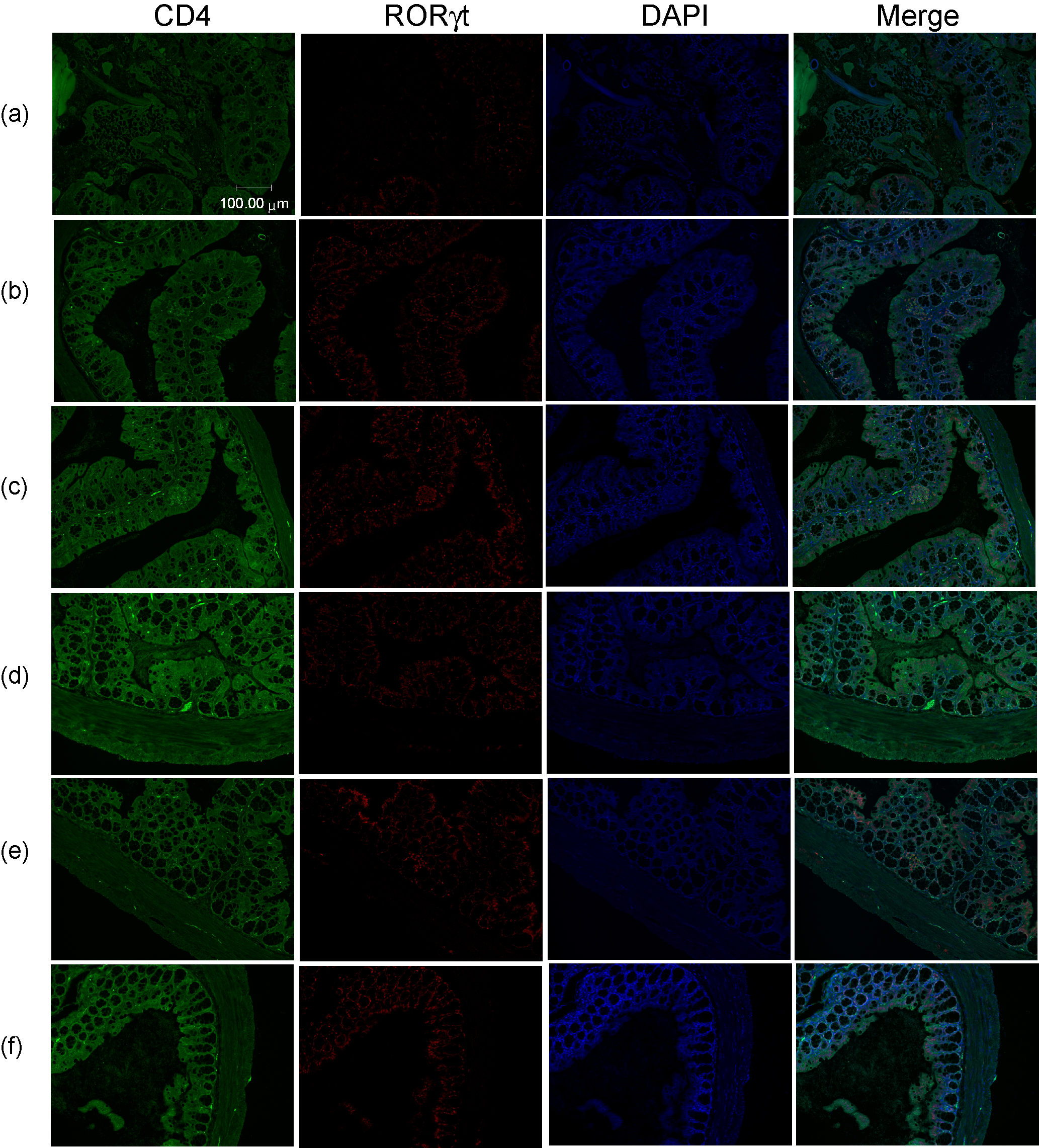

Supplement: S5 Fig — Images of colonic lamina propria after immunofluorescence staining using anti-CD4 antibody (green), anti-RORγt antibody (red), DAPI (blue) and combined fluorescence (merge) are observed with a fluorescence microscope. (a) 2-wk control, (b) 2-wk 1.5% EtOH, (c) 2-wk 5.0% EtOH, (d) 10-wk control, (e) 10-wk 1.5% EtOH, and (f) 10-wk 5.0% EtOH. (TIF) [file pone.0246580.s007.tif]

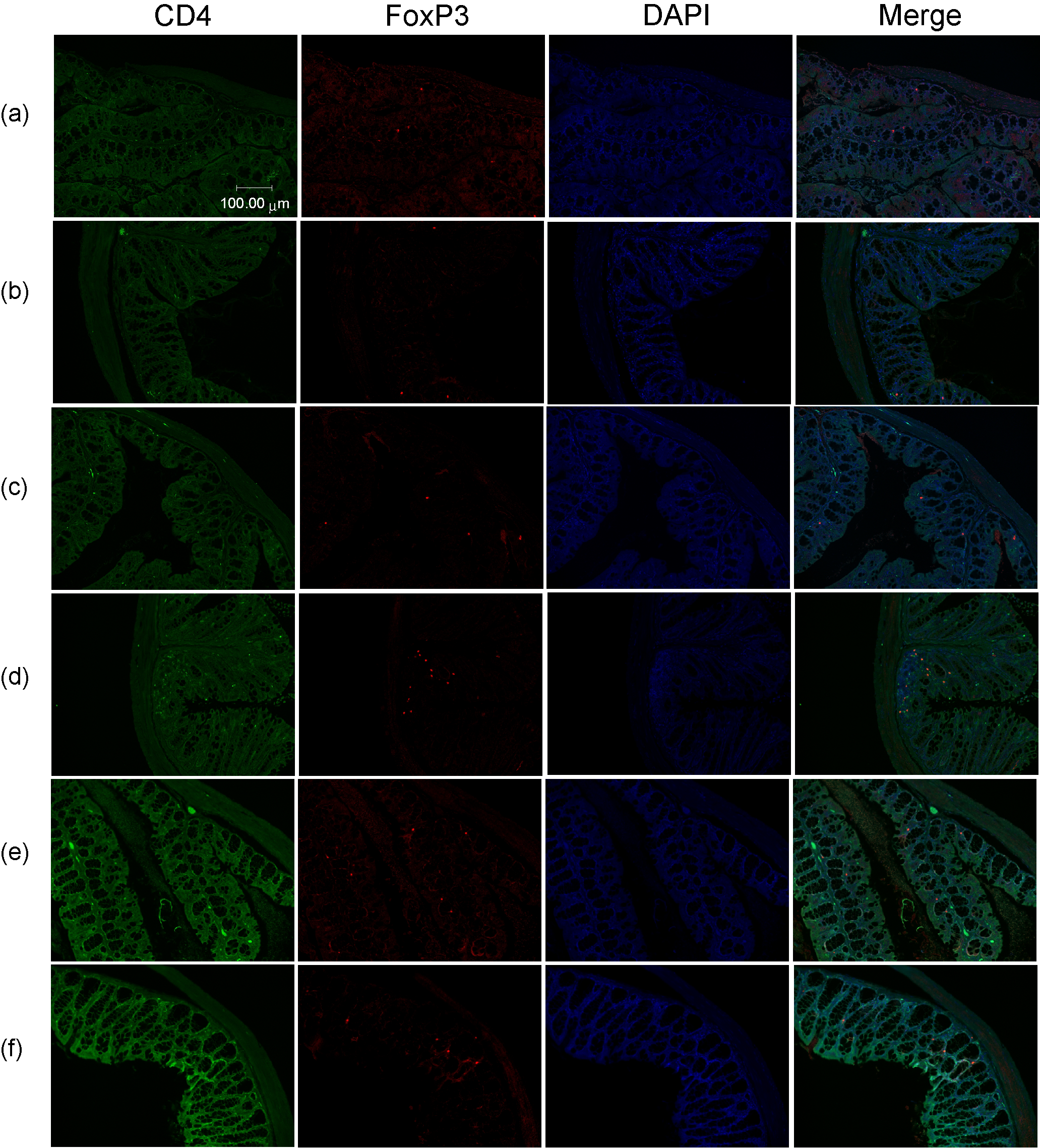

Supplement: S6 Fig — Images of colonic lamina propria after immunofluorescence staining using anti-CD4 antibody (green), anti-Foxp3 antibody (red), DAPI (blue) and combined fluorescence (merge) are observed with a fluorescence microscope. (a) 2-wk control, (b) 2-wk 1.5% EtOH, (c) 2-wk 5.0% EtOH, (d) 10-wk control, (e) 10-wk 1.5% EtOH, (f) 10-wk 5.0% EtOH. (TIF) [file pone.0246580.s008.tif]

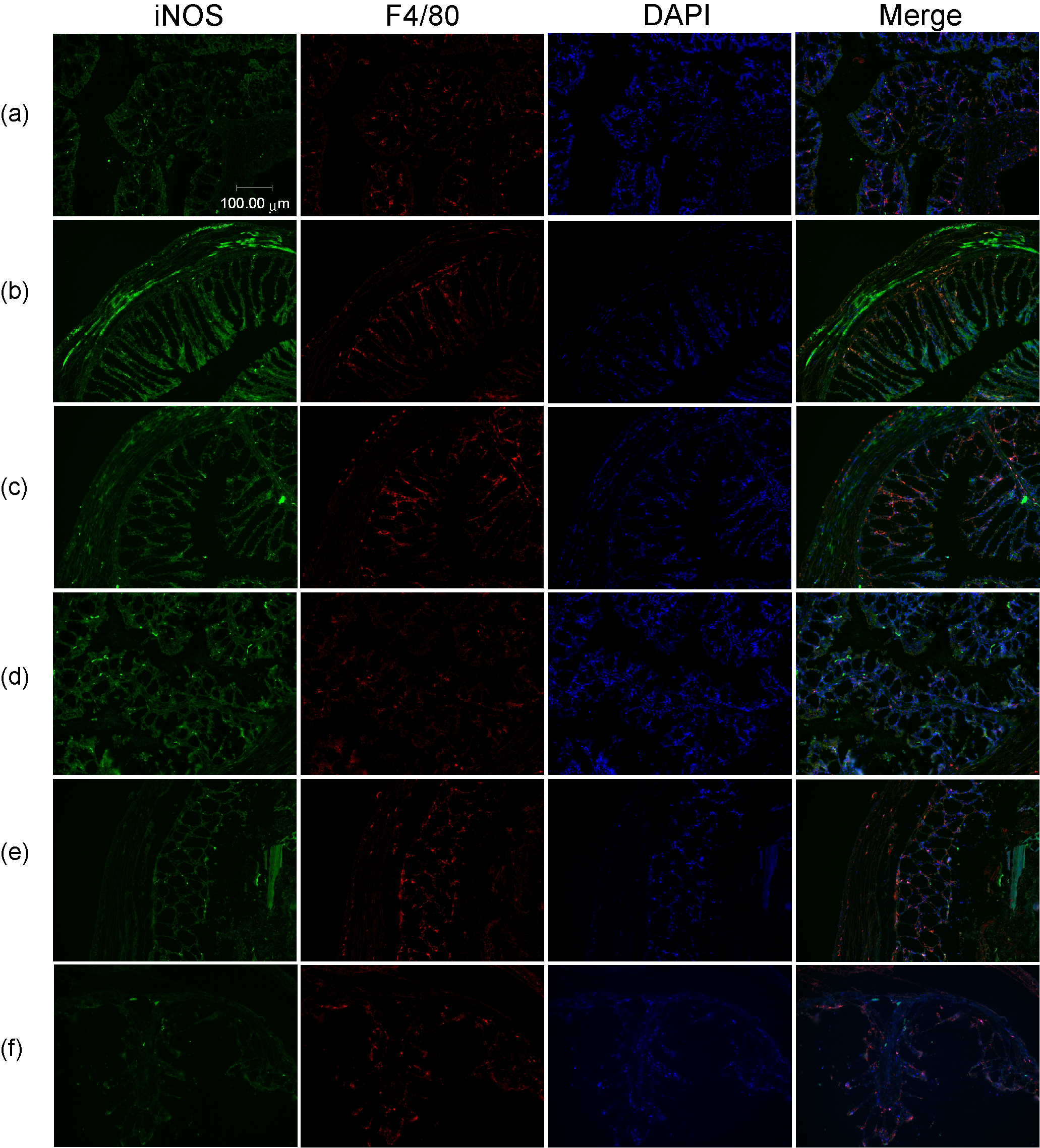

Supplement: S7 Fig — Images of colonic lamina propria after immunofluorescence staining using anti-iNOS antibody (green), anti-F4/80 antibody (red), DAPI (blue) and combined fluorescence (merge) are observed with a fluorescence microscope. (a) 2-wk control, (b) 2-wk 1.5% EtOH, (c) 2-wk 5.0% EtOH, (d) 10-wk control, (e) 10-wk 1.5% EtOH, (f) 10-wk 5.0% EtOH. (TIF) [file pone.0246580.s009.tif]

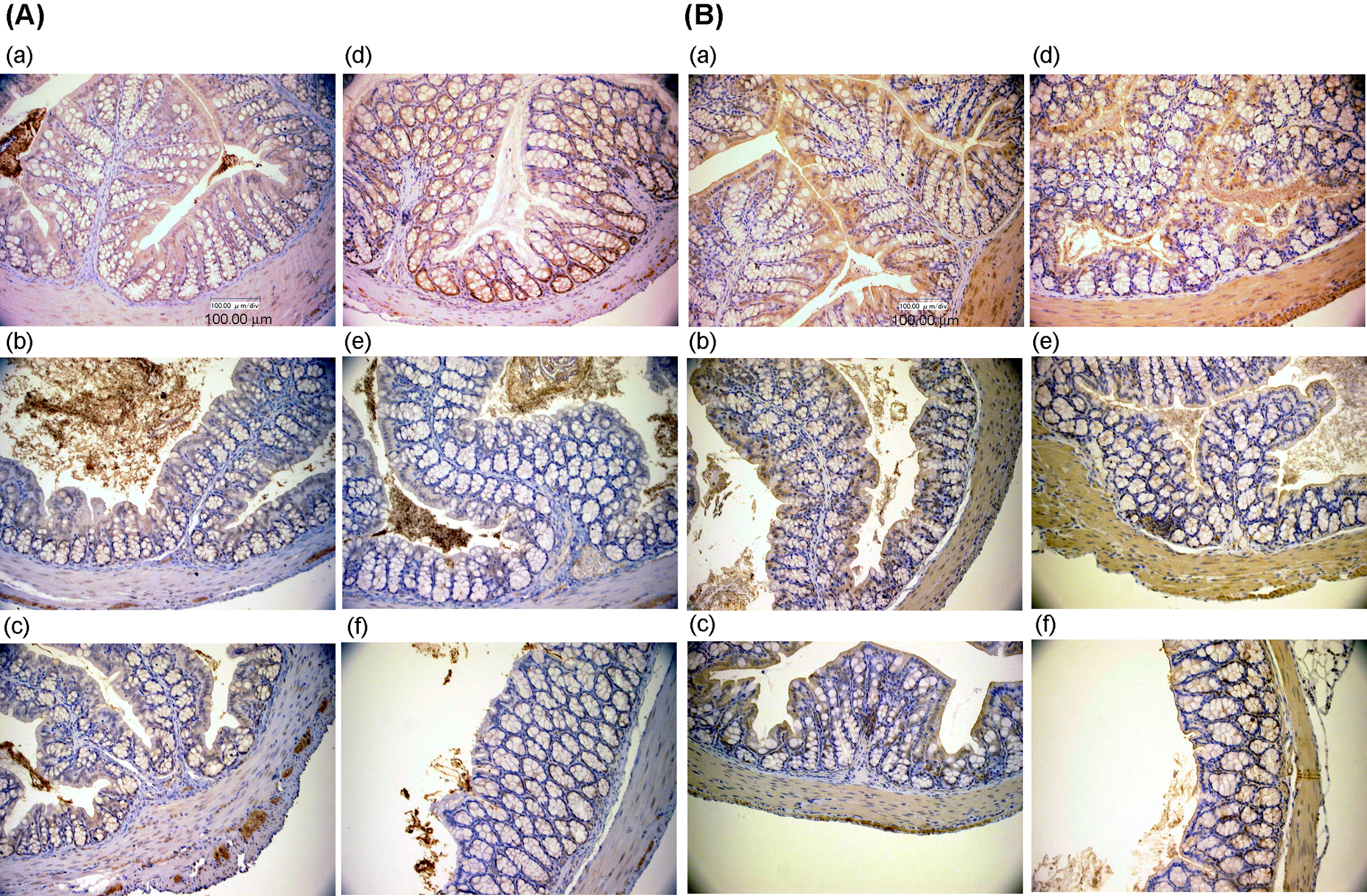

Supplement: S8 Fig — Images of colonic lamina propria after immunohistochemical staining using (A) polyclonal anti-AGEs antibody and (B) polyclonal anti-RAGE antibody are shown at 400X magnification. For abbreviations for ethanol-administration conditions, see the legend to Fig 2. For experimental details, see Materials and methods. (TIF) [file pone.0246580.s010.tif]
